# Structure of the *Legionella* Virulence Factor, SidC Reveals a Unique PI(4)P-Specific Binding Domain Essential for Its Targeting to the Bacterial Phagosome

**DOI:** 10.1371/journal.ppat.1004965

**Published:** 2015-06-12

**Authors:** Xi Luo, David J. Wasilko, Yao Liu, Jiayi Sun, Xiaochun Wu, Zhao-Qing Luo, Yuxin Mao

**Affiliations:** 1 Weill Institute for Cell and Molecular Biology and Department of Molecular Biology and Genetics, Cornell University, Ithaca, New York, United States of America; 2 Department of Biological Sciences, Purdue University, West Lafayette, Indiana, United States of America; Yale University School of Medicine, UNITED STATES

## Abstract

The opportunistic intracellular pathogen *Legionella pneumophila* is the causative agent of Legionnaires’ disease. *L*. *pneumophila* delivers nearly 300 effector proteins into host cells for the establishment of a replication-permissive compartment known as the *Legionella*-containing vacuole (LCV). SidC and its paralog SdcA are two effectors that have been shown to anchor on the LCV via binding to phosphatidylinositol-4-phosphate [PI(4)P] to facilitate the recruitment of ER proteins to the LCV. We recently reported that the N-terminal SNL (SidC N-terminal E3 Ligase) domain of SidC is a ubiquitin E3 ligase, and its activity is required for the recruitment of ER proteins to the LCV. Here we report the crystal structure of SidC (1-871). The structure reveals that SidC contains four domains that are packed into an arch-like shape. The P4C domain (PI(4)P binding of SidC) comprises a four α-helix bundle and covers the ubiquitin ligase catalytic site of the SNL domain. Strikingly, a pocket with characteristic positive electrostatic potentials is formed at one end of this bundle. Liposome binding assays of the P4C domain further identified the determinants of phosphoinositide recognition and membrane interaction. Interestingly, we also found that binding with PI(4)P stimulates the E3 ligase activity, presumably due to a conformational switch induced by PI(4)P from a closed form to an open active form. Mutations of key residues involved in PI(4)P binding significantly reduced the association of SidC with the LCV and abolished its activity in the recruitment of ER proteins and ubiquitin signals, highlighting that PI(4)P-mediated targeting of SidC is critical to its function in the remodeling of the bacterial phagosome membrane. Finally, a GFP-fusion with the P4C domain was demonstrated to be specifically localized to PI(4)P-enriched compartments in mammalian cells. This domain shows the potential to be developed into a sensitive and accurate PI(4)P probe in living cells.

## Author Summary

Legionnaires’ disease is caused by the intracellular bacterial pathogen *Legionella pneumophila*. Successful infection by this bacterium requires a special secretion system that injects nearly 300 effector proteins into the cytoplasm of host cells. The effector SidC and its paralog SdcA anchor on the *Legionella*-containing vacuole (LCV) and are important for the recruitment of ER proteins to the LCV. Recent data demonstrated that SidC and SdcA are ubiquitin E3 ligases and that their activity is required for the enrichment of ER proteins and ubiquitin conjugates on the LCV. Here we present the crystal structure of SidC revealing the architecture of a novel PI(4)P-binding module. Our biochemical and cell biological studies highlight key determinants involved in PI(4)P-binding and membrane insertion. Characterization of this novel PI(4)P binding module opens a potential avenue for the development of an accurate in vivo PI(4)P probe. Our data also reveals a distinct regulatory mechanism of the ubiquitin E3 ligase activity of SidC, which is activated by the lipid molecule, PI(4)P. Furthermore, our results suggest that proper spatial localization of SidC to the cytoplasmic surface of the bacterial phagosome through the binding with PI(4)P is crucial to its function.

## Introduction

The gram-negative bacterium *Legionella pneumophila* is ubiquitously found in natural water systems, where it is a parasite of free-living amoebae [1]. Upon inhalation of contaminated aerosols by susceptible individuals, this bacterium replicates within human macrophages and causes a severe pneumonia called Legionnaires’ disease [2]. During its interaction with host cells, *L*. *pneumophila* delivers approximately 300 effector proteins [3] into the host via its Dot/Icm (defective organelle trafficking/ intracellular multiplication) type IV secretion system [4,5]. Through the collective action of this large array of virulent proteins, *L*. *pneumophila* co-opts various host processes to facilitate bacterial survival and replication within the host. Among the many host cellular pathways, host membrane trafficking is one of the most studied processes manipulated by *L*. *pneumophila* [6].

Upon internalization, *L*. *pneumophila* forms a specialized membrane-bound compartment called the *Legionella*-containing vacuole (LCV). Previous data have shown that LCVs undergo a delicately programmed maturation process to evade fusion with lysosomes. The LCV is first covered by ER-derived vesicles at the early stage of its formation [7], and is later enriched with resident ER proteins [8,9] and then studded with ribosomes [10]. This biphasic maturation process converts the plasma membrane-derived LCV into an ER-like compartment and thus allows for the establishment of a replication-permissive niche within host cells [11]. The recruitment of ER and ER-derived vesicles requires sophisticated interactions between *Legionella* effectors and host factors. For example, Rab1, a small GTPase in the Rab family involved in vesicle trafficking between the ER and Golgi [12] has been shown to be recruited to the LCV by SidM (DrrA) [13,14]. Following Rab1 recruitment, a cascade of *Legionella* effectors, including SidM [15], AnkX [16], SidD [17,18], Lem3 [19], and LepB [20], regulate the spatial and temporal dynamics of Rab1 through canonical GEF and GAP activities, as well as protein posttranslational modifications [21].

Lipid molecules, particularly phosphoinositides (PIs) play crucial roles during the transition of LCVs into ER-like compartments [22]. PIs are a collection of lipids that have their inositol headgroup reversibly phosphorylated at the 3’, 4’, and 5’ positions. Although PIs are minor components of cellular membranes, they play fundamental roles in a broad range of cell signaling and membrane trafficking events [23]. One of the PI species, phosphotidylinositiol 4-phosphate (or PI(4)P) has been shown to accumulate on the LCV [24]. The enrichment of PI(4)P on the LCV is facilitated by both *Legionella* effectors and host PI metabolizing enzymes. SidF, the first *Legionella* protein shown to directly modify host PIs [25], anchors on the LCV and specifically hydrolyzes PI(3,4)P_2_ and PI(3,4,5)P_3_ to PI(4)P and PI(4,5)P_2_, respectively. Another *Legionella* encoded PI phosphatase, SidP, likely prevents the accumulation of PI(3)P on the LCV by hydrolyzing this lipid into phosphoinositol [26]. The *Legionella* effector protein LpnE appears to recruit the host PI-5-phosphatase OCRL to the LCV, which converts PI(4,5)P_2_ to PI(4)P [27]. Meanwhile, host phosphatidylinositol 4-kinases (PI4Ks) also play a role in the establishment of PI(4)P-enriched vacuoles [28,29]. Lipid remodeling on the LCV is critical for the selective anchoring of effectors to its surface. The specific recruitment of the Rab1 modulator SidM to the LCV is mediated by a C-terminal unique PI(4)P-binding P4M domain [28,30,31]. The recruitment of other LCV-localizing effectors, such as LidA [13] and LpnE [27] is likely also mediated by binding to PI(4)P.

Among the currently identified PI(4)P-binding *Legionella* effectors, the protein SidC and its paralog SdcA were shown to anchor on the LCV through a 20 kDa PI(4)P-binding domain called P4C (PI(4)P binding of SidC) to facilitate the recruitment of ER proteins to the LCV [32]. Deletion of *sidC* and *sdcA* resulted in a delay in the establishment of the replicative vacuole and a delayed appearance of ubiquitin signals on the LCV [33]. Recent structural studies of SidC revealed a novel N-terminal SNL domain that represents a unique family of ubiquitin E3 ligases [34]. Interestingly, ubiquitin ligase activity is required for the efficient recruitment of ER vesicles and ubiquitinated protein species to the LCV [34]. With the deciphering of the biochemical mechanisms of SidC, an intriguing question emerged as to how the P4C domain of SidC targets and likely regulates the E3 ligase activity of the SNL domain.

Here we report the crystal structure of SidC (aa 1–871) containing the SNL, the P4C. and a C-terminal undefined domain. Although the structure of the SNL domain is similar to the structure of the isolated SNL domain reported previously [33–35], significant conformational changes were observed. These include a hinge motion between the SNL and the insertion domains, as well as a rearrangement of residues near the catalytic triad of the ubiquitin ligase. We also show the structure of the P4C domain. Unlike any other known PI(4)P-binding domain, the P4C domain of SidC comprise a four α-helix bundle and the PI(4)P binding site resides in a highly positively charged pocket formed at one end of the bundle. Further analyses of the P4C domain both in vitro and in vivo shed light on the molecular mechanism of PI(4)P binding and LCV anchoring of SidC. Our data further provide a mechanistic background for the potential usage of the P4C domain as an accurate PI(4)P probe in general cell biology studies.

## Results

### Overall crystal structure of SidC

Full-length SidC from the Philadelphia 1 strain of *L*. *pneumophila* is a 106 kDa protein comprised of 917 residues [36]. we carried out structural studies of SidC.to provide insight into the molecular mechanisms of its biological functions, The N-terminal conserved SNL domain was crystallized and our structure-driven approach successfully showed that the SNL domain is a novel E3 ligase [34]. We extended our structural studies to full-length SidC. Although thin plate-shaped crystals were obtained, these crystals were poorly diffracted by X-ray beams. Crystals were also obtained with a truncated form of SidC (aa 1–871, from here on referred to as SidC871) in which 46 residues were deleted from the C-terminus. These crystals diffracted up to 2.9 Å and the structure was solved by the molecular replacement method using our previously determined SidC (aa 1–542, SidC542) crystal structure as the search model. Residues of the C-terminal portion of SidC871 were built de novo to the model interspersed with iterative refinement. The final refined model contains two molecules of SidC in the asymmetric unit and each molecule contains all residues from 8 to 864. The structure was refined to a resolution of 2.9 Å with R_work_/R_free_ = 22.7/28.1 and no significant stereochemistry violations (S1 Fig and S1 Table).

**Fig 1:**
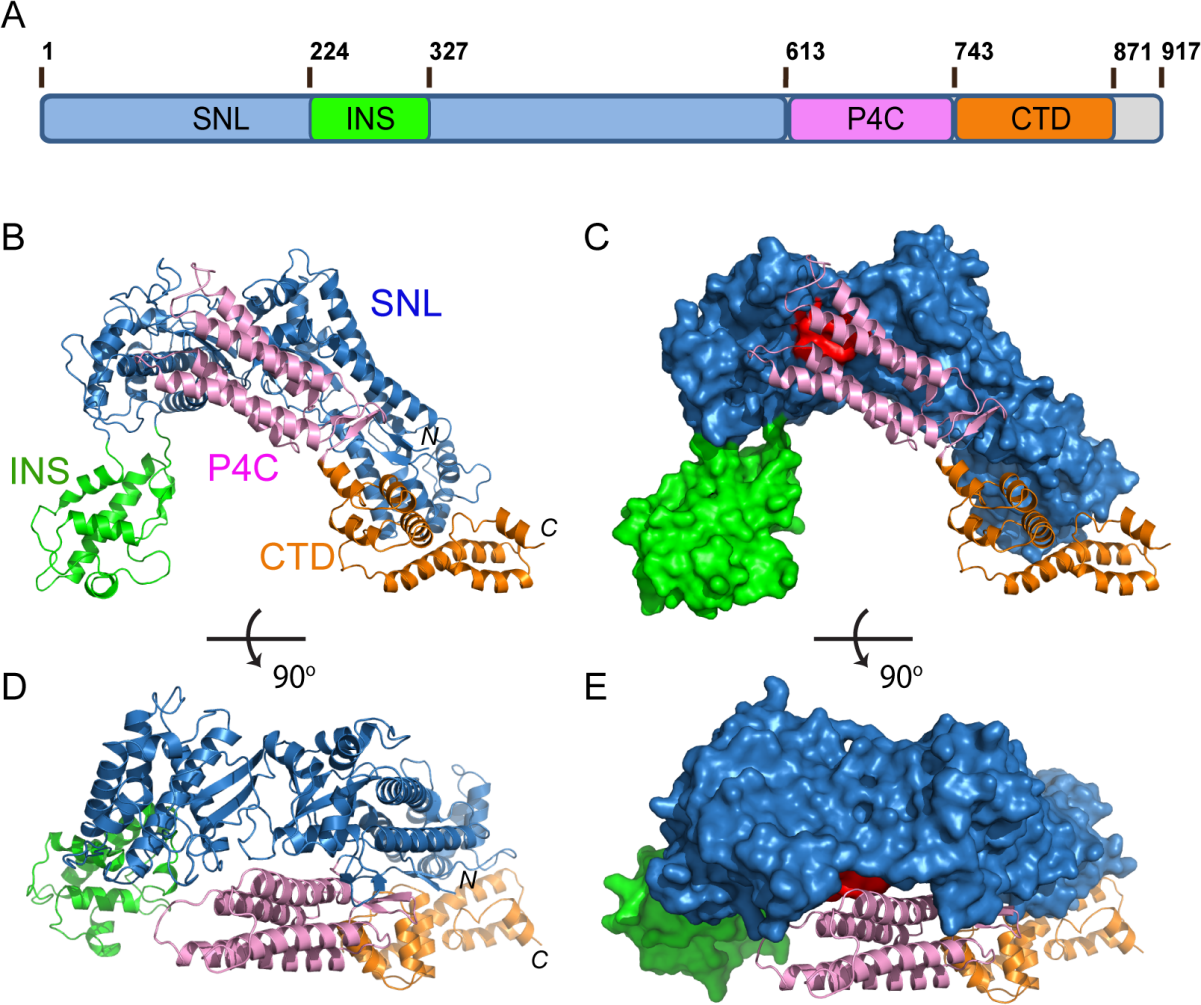
Crystal structure of SidC871 (aa. 1–871). (A) Schematic diagram of the domain structure of SidC. SidC contains an N-terminal SNL domain, an insertion domain (INS), a C-terminal PI(4)P-binding domain (P4C), and a C-terminal domain with unknown function (named CTD, colored in brown). (B) Ribbon diagram of the overall structure of SidC. (C) Overall structure of SidC with the SNL and INS domains shown in surface and the P4C and CTD in ribbon. The ubiquitin ligase active site in the SNL domain is colored in red. (C) and (B) have the same orientation. (D) A 90^0^ rotated view of (B). (E) A 90^0^ rotated view of (C). Color scheme from (A) to (E): the SNL domain is in blue; the INS domain is in green; the P4C domain is in pink; and the CTD domain is in brown.

The crystal structure of SidC871 reveals that SidC comprises four distinct domains: the N-terminal SNL domain, the INS domain (which is inserted within the SNL domain), the P4C domain, and a C-terminal unknown domain named CTD (Fig 1A–1E). These four domains are packed in a single arch-like shape with the P4C and the CTD domains in close contact with the SNL domain (Fig 1B–1E). Strikingly, the ubiquitin E3 ligase catalytic site (colored in red in Fig 1C and 1E) on the SNL domain is covered by the P4C domain. This observation suggests that the P4C domain is required to move away from the SNL domain in order to access the active site of the ubiquitin ligase. A plausible hypothesis is that the binding of PI(4)P by the P4C may cause the P4C domain to shift away from the catalytic site allowing the exposure of the ubiquitin ligase catalytic site. Testing of this hypothesis will be tested below.

### Conformational dynamics of the SNL and INS domains

**Fig 2:**
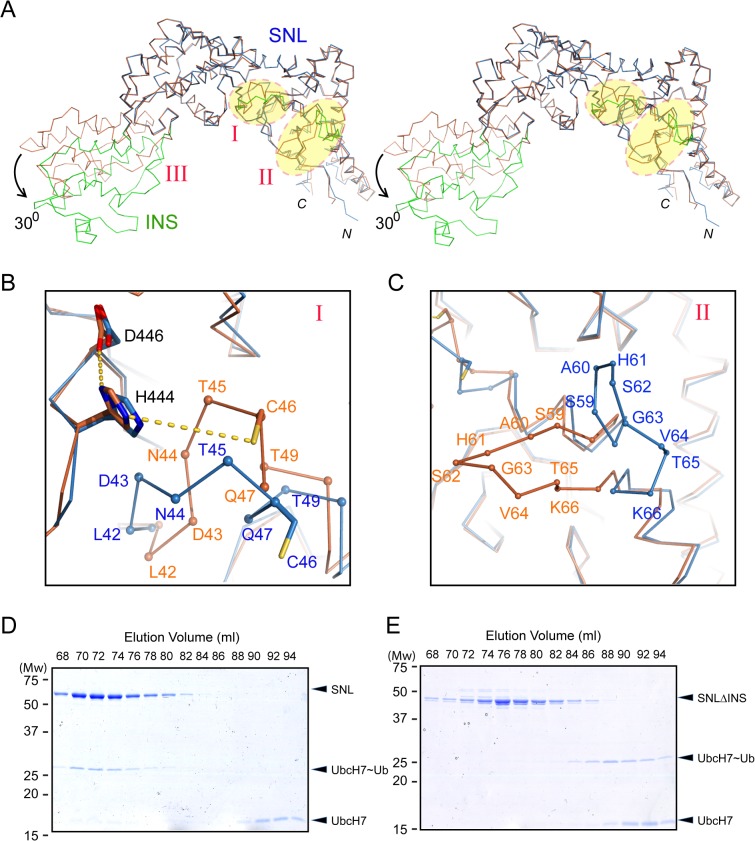
Conformational dynamics of the SNL and INS domains. (A) Stereo view of the Cα trace of the SNL (blue) and INS (green) domains of SidC in overlay with our previously reported SNL-INS domain structure (light brown; PDB ID: 4TRH). The three areas that have major conformational changes are labeled with I, II, and III, respectively. The INS domain in SidC871 is bent by about 30^0^ relative to the SNL domain. (B) Zoom-in view of the conformational changes at the catalytic site. C46 is shifted away from H444 and D446 in SidC871 (blue) compared with the SidC542 structure (brown). (C) Zoom-in view of the conformational change at the non-conserved loop (residue 59–66). (D) The SNL domain (SidC542) forms a stable complex with UbcH7~Ub. SDS gel of the size exclusion chromatography fractions from the sample containing SNL domain with ubiquitin-charged UbcH7. (E) The INS domain is involved in the binding of the SNL domain with UbcH7~Ub. SDS-gel of the size exclusion chromatography fractions from the sample containing the SNLΔINS domain with ubiquitin-charged UbcH7. UbcH7~Ub did not co-migrate with the SNLΔINS domain.

Structural comparison of the SNL and INS domains in SidC871 with the previously determined SidC542 structure, which contains only the SNL and INS domains [34] revealed three regions with major conformational changes (Fig 2A). The first area is the loop (residues 42–49) that contains the catalytic cysteine C46 (Fig 2A and 2B). In the SidC542 structure, C46 forms a catalytic triad with its nearby D446 and H444. However, C46 in SidC871 is shifted away from H444 by about 6 Å (Figs 2B and S1). This conformational switch involves large Psi-Phi angle changes of L42, T45, and C46 by the flipping of main chain peptide bonds (S2 Fig). The second region is a non-conserved loop (residue 59–66) on the interface between the SNL domain and the P4C domain (Fig 2C). The conformational change of this loop makes room for the P4C domain to pack against the SNL domain. However, the biological role of the structural flexibility of this loop is not clear.

The third major conformational change occurs at the two flexible linkers between the SNL and INS domains. The SNL domain is rotated along a hinge (residue S224 and S328) by about 30^0^ in SidC871 compared to the orientation in SidC542 (Fig 2A). This hinge bending motion is reminiscent of a similar displacement between the N- and the C-lobes of the HECT family ubiquitin ligases [37,38]. These observations imply that the INS domain may interact with E2s in the ubiquitin conjugating reaction. To test this hypothesis, we used size exclusion chromatography to analyze the interaction between E2 and SidC. The catalytically inactive C85S mutant UbcH7 was stably charged with ubiquitin (UbcH7~Ub) and incubated with SidC542. The protein mixture was then separated by size exclusion chromatography and elution fractions were analyzed by SDS-PAGE. UbcH7~Ub, but not UbcH7 alone was able to form a stable complex with SidC542 as indicated by the co-fractionation of SidC542 with UbcH7~Ub, but not with UbcH7. In contrast, although the SidC542 ΔINS mutant behaved well as a mono-dispersed protein [39], it failed to interact with Ub-UbcH7 (Fig 2E). These data suggest that the INS domain mediates the binding with E2s. Furthermore, like the E2~Ub-HECT E3 ternary complex [40], the covalently linked ubiquitin likely interacts with an area near the E3 catalytic site, thus stabilizing the E2~Ub-SidC ternary complex. Consistent with these results, the SidC542 ΔINS mutant, which failed to bind E2, is completely inactive in ubiquitination assays (S3 Fig).

### A novel highly specific PI(4)P-binding domain

**Fig 3:**
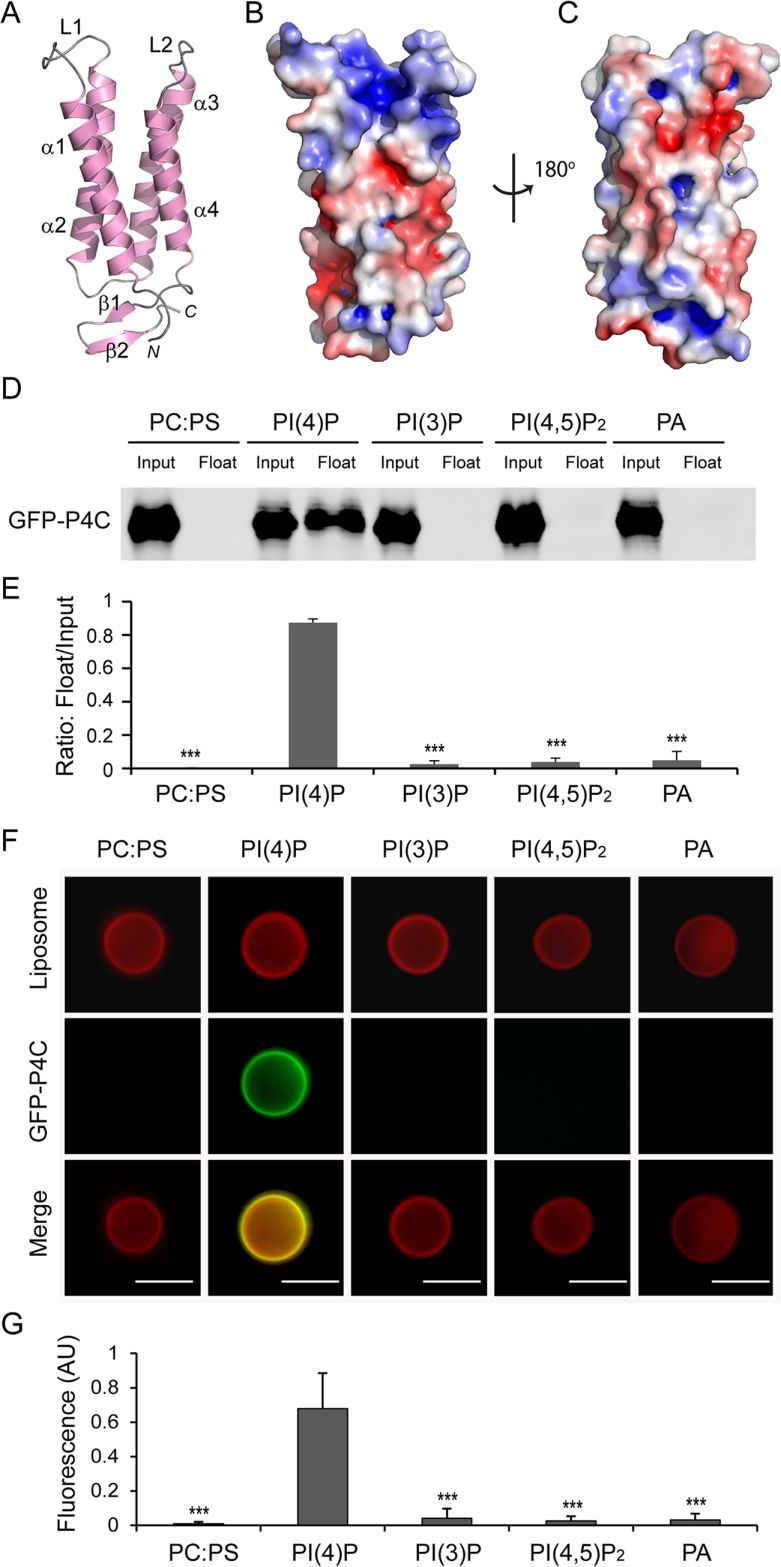
The P4C domain of SidC specifically binds with PI(4)P. (A) Ribbon diagram of the P4C domain. (B) Molecular surface of the P4C domain. The surface is colored based on electrostatic potential with positively charged region in blue (+5 kcal/electron) and negatively charged surface in red (-5 kcal/electron). (C) Back view of the surface model of the P4C domain. (D) Liposome floatation assays. Input and float samples were analyzed by SDS-PAGE and immunoblotted with anti-GFP antibodies. Recombinant GFP-P4C showed selective binding to PI(4)P-positive liposomes but not the liposomes with other components. (E) Quantification of liposome floatation assays from three independent experiments. Error bars represent standard deviation. (F) Fluorescent images of liposome binding by GFP-P4C. Only the liposomes containing PI(4)P showed strong binding of GFP-P4C. Scale bar = 10 μm (G) Quantification of liposome binding of GFP-P4C. GFP fluorescent signals were normalized to red Dil dye signals on the same liposome and averaged on three randomly picked liposomes. Error bars represent standard deviation. ** *p* < 0.01; *** *p* < 0.001.

SidC contains a specific PI(4)P-binding domain that lies between residues 609–776 and this 20 kDa region was named P4C (PI(4)P binding of SidC) [32]. Our crystal structure of SidC871 revealed the architecture of this unique domain. The P4C domain (aa. 614–743) mainly comprises four α-helices running in an antiparallel way. These four α-helices form a bundle with one end sealed by a C-terminal short β hairpin. A pocket is created at the other end by two loops (L1 and L2) connecting α1, α2 and α3, α4, respectively (Fig 3A).The calculated electrostatic surface potentials revealed that this pocket is highly positively charged (Fig 3B) and it is the only area clustered with positive charges in the domain (Fig 3C). Our structural observations suggest that the positively charged pocket is likely the binding site for phosphoinositides (PI).

To test the specificity of PI binding, we performed liposome floatation assays [41]. Liposomes were prepared with 80% phosphatidylcholine (PC), 10% Phosphatidylserine (PS) and 10% either PI(4)P or other phospholipids. After incubation with purified GFP-tagged P4C proteins for 30 min, a sucrose gradient centrifugation was performed to separate liposomes from unbound proteins. The floated liposomes were pooled and normalized, and bound proteins were analyzed by Western blot followed by densitometry quantification. A large number of GFP-P4C proteins were recovered in the liposome samples containing PI(4)P. However, almost no GFP-P4C co-floated with liposomes in the absence of PI(4)P or with liposomes containing either PI(3)P, PI(4,5)P_2_, or phosphatidic acid (PA) (Fig 3D and 3E). The specific interaction with PI(4)P by the P4C domain can also be directly visualized by fluorescence microscopy. Large unilamellar vesicles (LUV) were prepared with lipid compositions similar to those used in the liposome floatation assays. In addition, a Dil dye (Invitrogen) was incorporated into the LUV for fluorescence detection. After a 30 min incubation of GFP-P4C with the liposomes, the samples were imaged under a fluorescence microscope. In agreement with the results from the liposome floatation assay, strong GFP signals were observed on the surface of the liposomes containing PI(4)P, whereas there was almost no detectable GFP signal for the liposomes without PI(4)P (Fig 3F and 3G). Together, these results demonstrate that the P4C domain of SidC binds to PI(4)P with a high selectivity.

### Determinants of PI(4)P recognition and membrane targeting by the P4C domain

**Fig 4:**
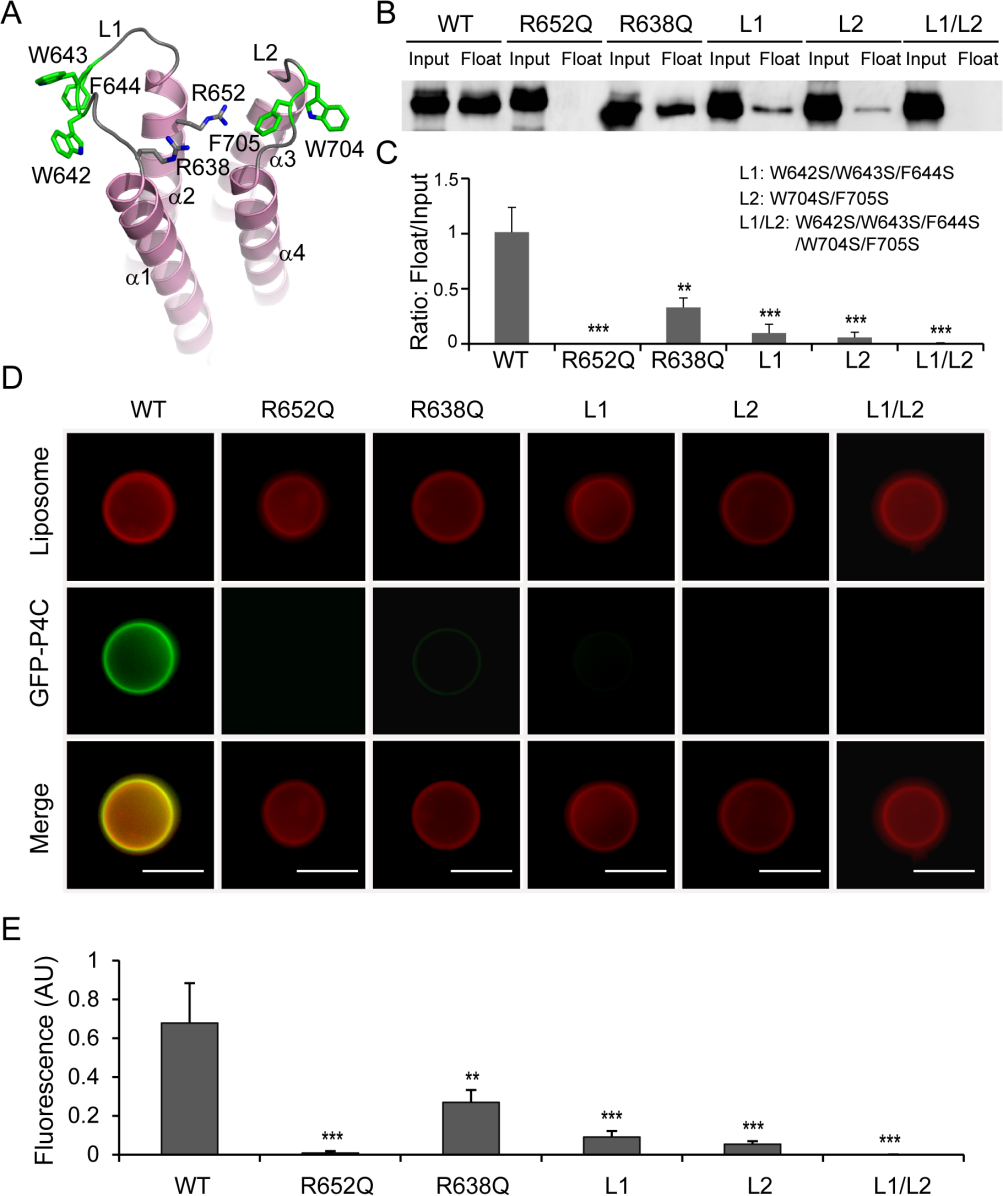
Determinants of PI(4)P recognition and membrane targeting by the P4C domain. (A) Ribbon diagram of the P4C domain. Residues that play a role in membrane interaction are highlighted in sticks. R652 and R638 form a pocket for the binding of the PI(4)P headgroup. The L1 (W642, W643, and F644) and L2 (W704 and F705) loops are colored in green, and form the membrane interacting motif (MIM). (B) Liposome floatation assay for P4C mutants. The R652Q and the L1/L2 (W642S/W643S/F644S/W704S/F705S) mutants completely abolished PI(4)P- liposome binding. (C) Quantification of liposome floatation assays of P4C mutants averaged from three independent assays. (D) Fluorescent images of liposome binding by GFP-P4C mutants. Mutations of cationic residues in the PI(4)P binding pocket and hydrophobic residues at the two membrane insertion loops significantly reduce the binding to PI(4)P-containing liposome. Scale bar = 10 μm. (E) Quantification of liposome binding of GFP-P4C mutants. GFP fluorescent signals were normalized to red Dil dye signals on the same liposome and averaged on three randomly picked liposomes. Error bars represent standard deviation. ** *p* < 0.01; *** *p* < 0.001.

Membrane association by PI-binding proteins is generally governed by the stereospecific recognition of a distinct PI headgroup and the nonspecific insertion of hydrophobic residues into the membrane bilayer [42,43]. The structure of the P4C domain highlights these key features for its association with PI(4)P-containing membranes. First, there are two conserved arginine residues (R638 and R652) located in the positively charged pocket of the P4C domain (Figs 4A and S4). These two arginine residues contribute to the overall positive charge of the pocket and may be directly involved in PI(4)P binding by making hydrogen bonds and salt bridges with the headgroup of PI(4)P. To test this hypothesis, we constructed mutants in which each of the two arginine residues was replaced with a glutamine. The R638Q mutation significantly reduced the binding to PI(4)P-containing liposomes to ~30% of that of the wild type P4C, while the R652Q mutation nearly completely abolished the PI(4)P binding by the P4C domain (Fig 4B and 4C). The second feature is the hydrophobic nature of the two loops L1 and L2 that surround the PI(4)P binding pocket (Fig 4A). L1 contains three consecutive hydrophobic residues, W642, W643, and F644 while L2 contains two, W704 and F705. Although these residues are variable, their hydrophobic nature is conserved (S4 Fig). These hydrophobic residues likely function as a “membrane insertion motif” (MIM). Mutation of these hydrophobic residues to the polar residue serine either in L1 (W642S/W643S/F644S) or in L2 (W704S/F705S) significantly reduced the binding to PI(4)P-containing liposomes, whereas the binding was completely abolished when all five hydrophobic residues were mutated (Fig 4B and 4C). In agreement with the floatation assays, the effects of P4C mutations on liposome binding were further confirmed by direct fluorescence imaging (Fig 4D and 4E). These data demonstrate that the high affinity PI(4)P binding by the P4C domain of SidC requires both cationic residues, which mediate the recognition of the headgroup of PI(4)P, and the MIM, which enhances membrane association by direct insertion of hydrophobic side chains into the membrane bilayer.

### Intracellular localization of the P4C domain in living cells

**Fig 5:**
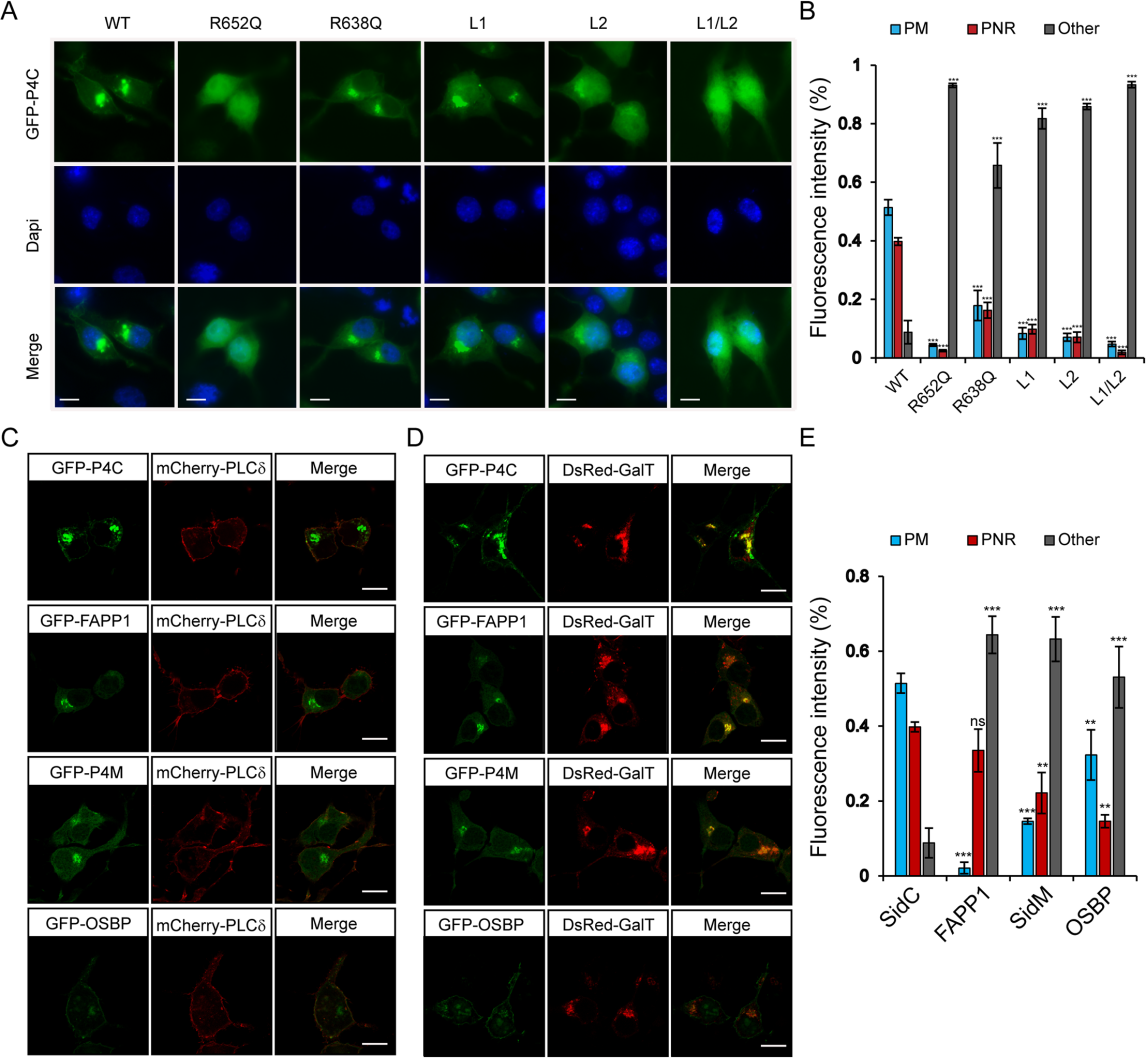
Intracellular localization of fluorescent protein fusions of the P4C domain from SidC. (A) GFP-tagged wild type P4C domain localized to the perinuclear region and plasma membrane, while this localization is altered in PI(4)P-binding defective P4C mutants in N2A cells. The nucleus was stained with DAPI. Wild type P4C showed both plasma membrane and perinuclear localization. The R638Q, L1, and L2 mutants had a more diffuse localization while the R652Q and the L1/L2 mutants were completely cytosolic. (B) Quantification of the intracellular localization of GFP-P4C represented by the percentage of the fluorescence intensities at the plasma membrane (PM), perinuclear region (PNR), and other areas of the cell. Error bars represent standard deviation. The measurements were averages of three randomly selected cells. (C) Confocal images of localizations of the plasma membrane marker mCherry-PLCδ-PH with GFP-tagged PI(4)P probes in N2A cells. (D) Confocal images of colocalizations of the Golgi marker DsRed-GalT with GFP-tagged PI(4)P probes in N2A cells. Scale bar = 10 μm in all images. (E) Quantification of the intracellular localization of PI(4)P probes. Error bars represent standard deviation. ** *p* < 0.01; *** *p* < 0.001.

We further examined the specific PI(4)P binding by the P4C domain in living cells. Wild type and mutant P4C domains tagged with EGFP were expressed in N2A cells by transient transfection (Fig 5A). Wild type GFP-P4C displayed enrichment at both the plasma membrane (PM) and the perinuclear region, presumably the Golgi complex, which are the two major PI(4)P-rich compartments in the cell [44]. Consistent with the in vitro liposomal binding results, the R652Q mutant showed a cytosolic and nuclear localization, suggesting PI(4)P binding is severely hampered by this mutation. The R638Q, which showed a reduced affinity for PI(4)P in vitro, displayed a similar localization compared to the wild type but with a higher cytosolic distribution. Individual MIM mutations, L1 and L2, also displayed a high level of cytosolic localization, indicating a reduced affinity for the membrane. Strikingly, the L1 and L2 double mutation completely abolished the membrane localization of P4C, rendering the mutant a completely cytosolic protein (Fig 5A and 5B). Similar intracellular localization of wild type and mutant GFP-P4C domains was also observed in other cell lines, such as Cos7 (S5 Fig). These data further validate the key determinants for the binding of the P4C domain to PI(4)P-enriched membranes, suggesting a potential application of this domain as a general PI(4)P probe in living cells.

To explore the possibility of developing a new PI(4)P probe, we compared the PI(4)P binding and intracellular localization of the P4C domain with other reported PI(4)P probes. GFP-tagged PH domains of FAPP1 and OSBP [44,45] and the GFP-tagged P4M domain from the *Legionella* effector protein SidM/DrrA [30,31,46] were expressed and purified from *E*. *coli*. The binding of PI(4)P was analyzed with liposome floatation assays (S6 Fig). In the floatation experiments, a higher percentage of GFP-P4C co-floated with PI(4)P-containing liposomes than any of the other PI(4)P probes tested (S6A and S6B Fig). This observation was further supported by direct visualization of the binding with fluorescence microscopy (S6C and S6D Fig).

We then compared the intracellular localization of these PI(4)P probes in living cells. Constructs expressing GFP-tagged probes were co-transfected with either the PM marker, mCherry-PLCδ-PH [47] or the Golgi marker, DsRed-GalT [48] in N2A cells. The GFP-P4C probe displayed a significant co-localization with both the PM marker and the Golgi marker (Fig 5C–5E), indicating that the P4C domain is able to faithfully report the two major pools of PI(4)P in living cells. Although all three of the other probes also co-localized with GalT at the Golgi apparatus, their PM signals were weaker compared to those of P4C. In summary, our data suggest that the P4C probe from SidC has the potential to be developed as a more sensitive and unbiased biosensor for detecting PI(4)P in living cells.

### The interface between the P4C and SNL domain

**Fig 6:**
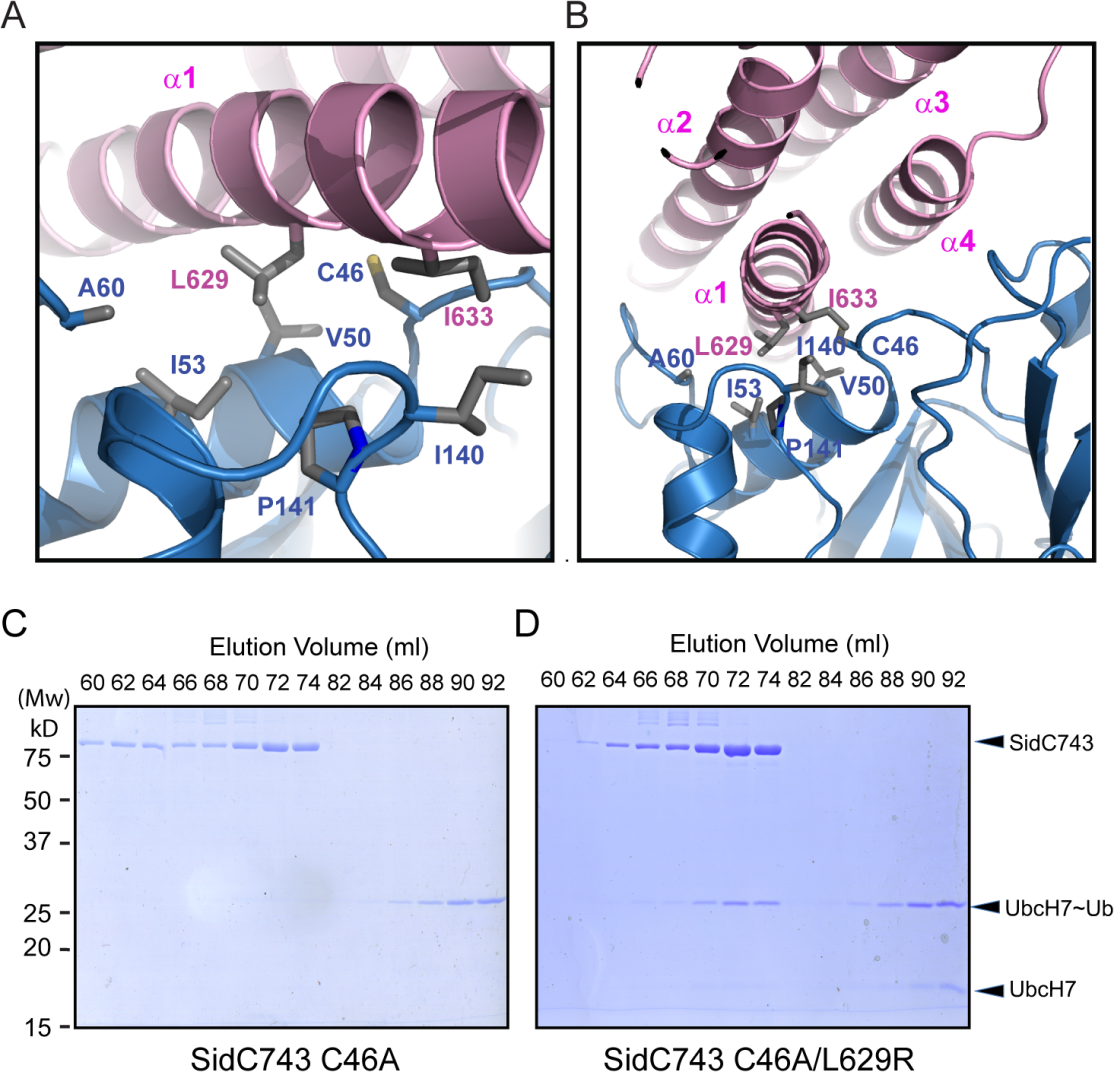
The interface between the P4C domain and the SNL domain. (A) and (B) Two orthogonal views of the interface between the P4C and SNL domains. Hydrophobic residues at the interface are shown in sticks. The P4C domain is colored in pink and the SNL domain in blue. (C) SidC743 C46A does not form a stable complex with ubiquitin-charged UbcH7, as indicated by SDS-PAGE gel analysis of fractions from size exclusion chromatography experiment. (D) SidC743 C46A/L629R forms a stable complex with UbcH7~Ub as demonstrated by the co-migration of SidC743 C46A/L629R with UbcH7~Ub on the size exclusion column.

Our crystal structure revealed that the P4C packs against the SNL domain and blocks the accessibility of the E3 catalytic site (Fig 1). The interaction between the P4C and SNL domains is mainly mediated by extensive hydrophobic interactions between residues L629 and I633 of the P4C domain and a hydrophobic patch consisting of residues V50, I53, A60, I140, and P141 near the ligase catalytic site on the SNL domain (Fig 6A and 6B). To characterize the nature of this intramolecular interaction, we created the L629R mutant with the speculation that the bulky and charged arginine residue would destroy both surface complementarity and the hydrophobic nature at the interface. Thus, this mutation would displace the P4C domain and keep SidC in an open conformation. Indeed, SidC743 C46A/L629R, but not SidC743 C46A, formed a stable complex with UbcH7~Ub as shown by size exclusion chromatography analysis (Fig 6C and 6D). The formation of the ternary complex is likely due to the open conformation caused by this mutation, which allows for the binding of the ubiquitin moiety to the catalytic site. These data suggest that the intramolecular interaction between the P4C and the SNL domain is not due to crystal packing. Instead, it likely represents an intramolecular regulatory mechanism in which the displacement of the P4C domain from the catalytic site switches the enzyme into an open conformation to release the occlusion at the ligase active site of SidC.

### Binding of PI(4)P by the P4C domain stimulates the ubiquitin E3 ligase activity of SidC

**Fig 7:**
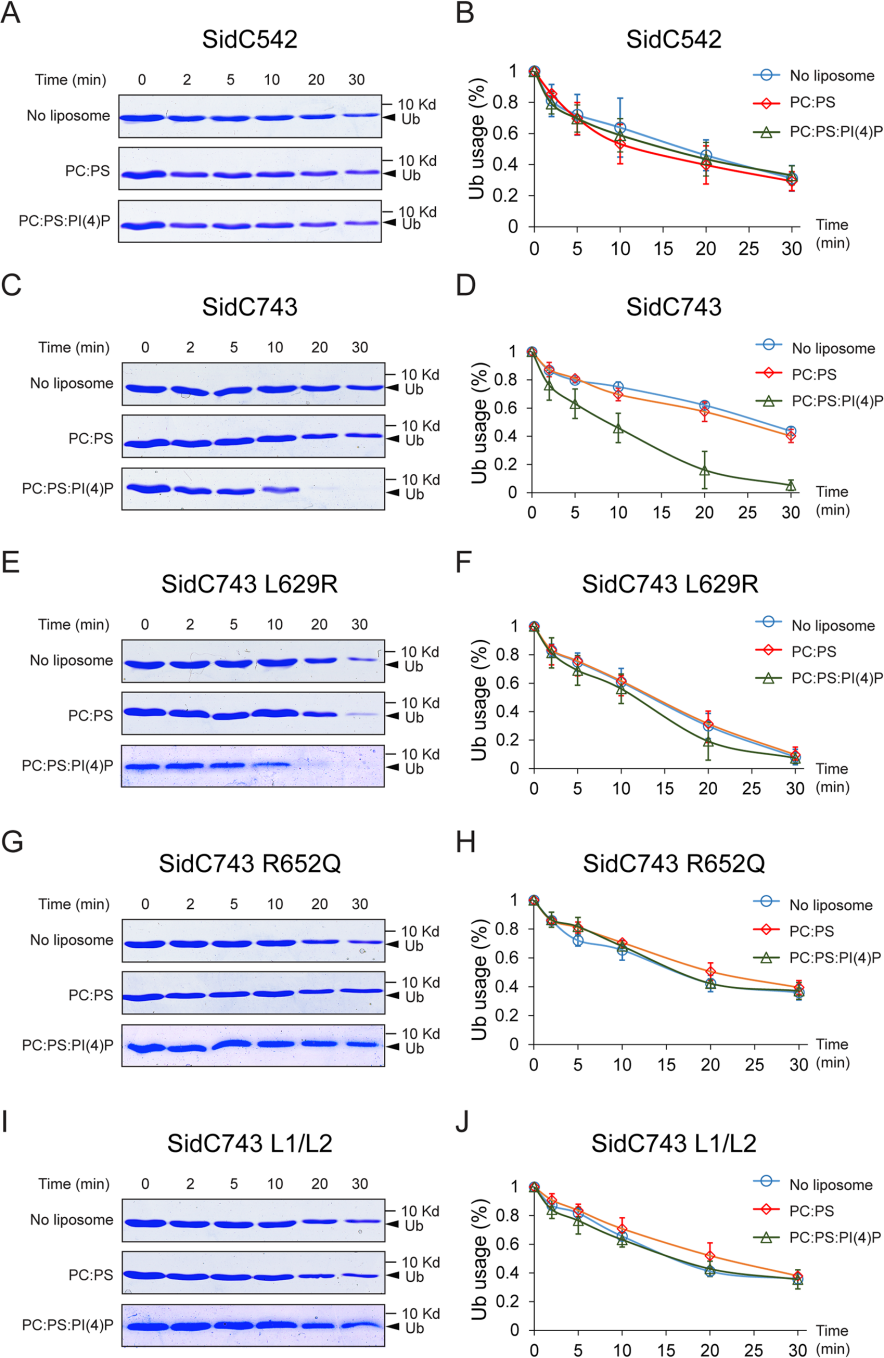
PI(4)P stimulates the ubiquitin E3 ligase activity of SidC. (A) In vitro ubiquitin ligase activity assay with SidC542 in the absence of liposomes and in the presence of liposomes containing PC/PS or PC/PS/PI(4)P. The reactions were stopped at the indicated time points and the samples were analyzed by SDS-PAGE. The decreased intensity of ubiquitin bands indicates the consumption of free ubiquitin during the ligase reaction. (B) Percentage of free ubiquitin left in the reaction at each indicated time points averaged from three independent experiments. (C) and (D) In vitro ubiquitin ligase activity assay with SidC743. The ubiquitin ligase activity is enhanced in the presence of PI(4)P. (E) and (F) In vitro ubiquitin ligase activity assay with SidC743 L629R. This mutant has higher ubiquitin ligase activity even in the absence of PI(4)P, presumably due to the open conformation caused by this mutation. (G)-(H) and (I)-(J) In vitro ubiquitin ligase activity assay with SidC743 R652Q andSidC743 L1/L2. No stimulation of the ubiquitin ligase activity by PI(4)P was observed with these two PI(4)P-binding defective mutants.

The intramolecular interaction between the P4C domain and the SNL domain led us to hypothesize that the P4C domain plays a role in regulating the E3 ligase activity. To test this model, we performed ubiquitin ligase activity assays and quantified the reaction by measuring the rate of ubiquitin consumption. SidC542, which lacks the C-terminal P4C domain, showed a similar ubiquitin ligase activity in the absence of liposomes or in the presence of liposomes containing PC/PS or PC/PS/PI(4)P (Fig 7A and 7B). However, SidC743, which contains the P4C domain, exhibited enhanced ligase activity in the presence of PI(4)P-containing liposomes (Fig 7C and 7D). These data suggest that the binding of PI(4)P may induce a conformational switch of the enzyme to an open form that exposes the catalytic site and stimulates the ligase activity. This conclusion is further supported by the observation that SidC743 L629R, which prefers an open conformation as shown above (Fig 6), is more active even in the absence of PI(4)P-containing liposomes compared to SidC743 (Fig 7C–7F). However, the stimulating effect caused by PI(4)P is abolished in both SidC743 R652Q and SidC743 L1/L2 mutants, which are defective in PI(4)P binding (Fig 7G–7J). In conclusion, PI(4)P-binding stimulates the ubiquitin ligase activity of SidC presumably through an induced conformational switch from a closed to an open form of the enzyme.

### PI(4)P-binding by the P4C domain is required for the localization of SidC to the *Legionella* containing vacuole (LCV)

Our structural and biochemical studies revealed the key determinants of the P4C domain for PI(4)P binding. We next asked whether these key residues are also responsible for the association of SidC with the LCV during bacterial infection. SidC and three SidC mutants (R652Q; L1/L2, which carries mutations of the MIM motif, W642S/W643S/F644S and W704S/W705S; and L1/L2/R652Q, which bears both R652Q and the MIM mutations) were constructed in the SidC-expressing plasmid pZL199 [49] and were transformed into the *L*. *pneumophila* strain Lp02 lacking both endogenous *sidC* and *sdcA*(Δ*sidC-sdcA*) [34,49]. All mutant proteins were expressed at a level comparable to the wild type (S7A Fig) and had no distinguishable difference in Dot/Icm-mediated translocation by the bacteria (S7B Fig). In agreement with in vitro results, the number of SidC-positive LCVs was reduced from more than 90% for the wild type to about 20% for both the R652Q and L1/L2 mutants (Figs S8A and 8B). Moreover, the average intensity of the immunofluorescent signals observed from the LCVs positive for PI(4)P binding-defective SidC mutants was about 10 times weaker than those from the wild type (S9A and S9B Fig). These data suggest that both the headgroup binding mediated by R652 and the membrane insertion mediated by the L1 and L2 loops are indispensable for anchoring SidC to the bacterial phagosome.

**Fig 8:**
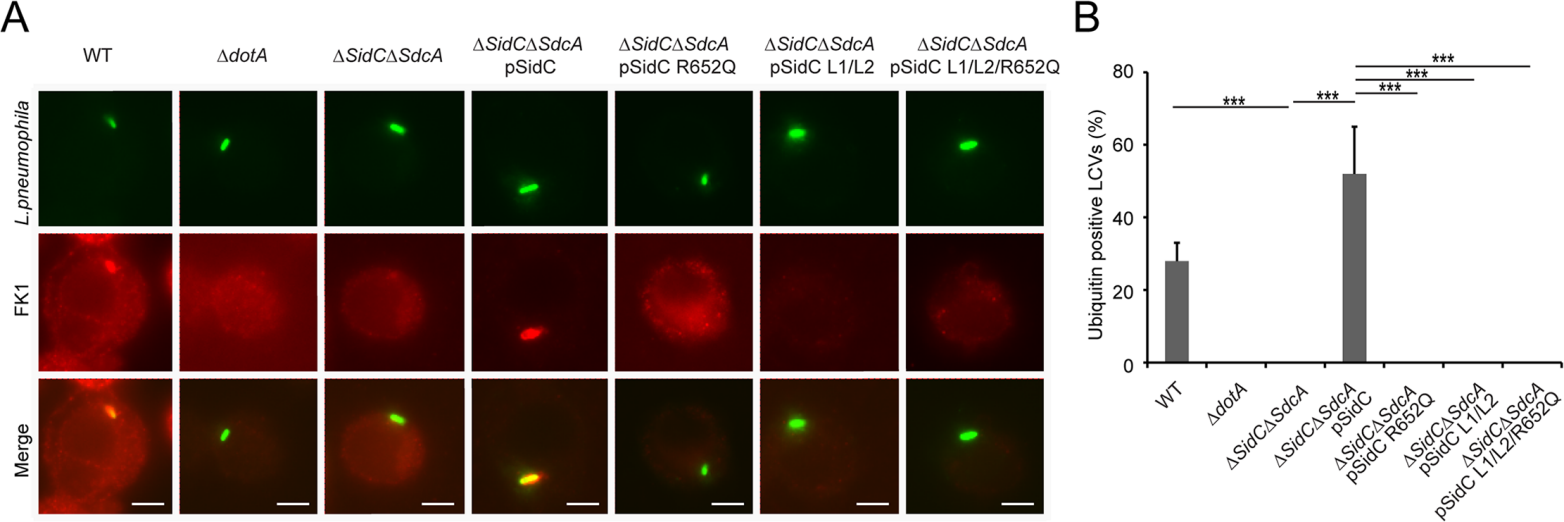
PI(4)P-binding by the P4C domain is essential for the recruitment of ubiquitinated species to the LCV. (A) Immuno-fluorescent staining of ubiquitinated species on the LCV. U937 Cells were infected with indicated *L*. *pneumophila* strains at an MOI of 1 for 2hrs and samples were fixed prior to immunostaining with antibodies against *Legionella* or m-ubiquitin (FK1). Strains: WT: *L*. *pneumophila* Philadelphia-1 strain Lp02; *dotA*: the Dot/Icm type IV secretion system defective strain Lp03; Δ*sidC-sdcA*: the *SidC* and *SdcA* double deletion mutant of the Lp02 strain; Δ*sidC-sdcA*(pSidC), Δ*sidC-sdcA*(pSidC R652Q), Δ*sidC-sdcA*(pSidC L1/L2), and Δ*sidC-sdcA*(pSidC L1/L2/R652Q): Δ*sidC-sdcA* strain complemented with a plasmid expressing either wild type or PI(4)P binding-defective mutants. (B) Percentage of cells containing ubiquitin positive LCVs counted from three independent experiments (at least 150 vacuoles were scored in each experiment). ** *p* < 0.01; *** *p* < 0.001.

### PI(4)P binding by the P4C domain is essential for the recruitment of ubiquitin and ER proteins to the LCV

Shortly after *L*. *pneumophila* infection, polyubiquitin conjugates are recruited to the bacterial phagosome [50]. We have recently shown that the ubiquitin E3 ligase activity of SidC, which is mediated by its N-terminal SNL domain, is required for this recruitment [34]. Here we ask whether the recruitment of ubiquitinated species to the LCV is dependent on the P4C domain-mediated anchoring of SidC to the LCV. U937 macrophages were infected with *Legionella* strains expressing either wild type or SidC mutants. Ubiquitin signals were detected by immunostaining with the FK1 antibody. This recruitment was almost abolished in infections with the Δ*sidC-sdcA* mutant strain, and wild type SidC completely restored the recruitment. However, none of the mutants defective in PI(4)P binding retained this function (Fig 8A and 8B). Thus, the association of SidC with the LCV via the P4C domain is necessary for the recruitment of ubiquitinated species.

SidC and its paralog SdcA are also important for efficient recruitment of ER proteins to the bacterial phagosome [32]. Using a *Dictyostelium discoideum* strain stably expressing GFP-HDEL [51] (a GFP fusion with the ER retention marker HDEL), we have shown that the ubiquitin E3 ligase activity of SidC plays a critical f role in the recruitment of ER components to the bacterial phagosome [34]. To address whether the recruitment of ER components is also dependent on the P4C domain-mediated anchoring of SidC to the LCV, *D*. *discoideum* cells stably expressing GFP-HDEL were infected with relevant *Legionella* strains. In *D*. *discoideum* infected with wild type *L*. *pneumophila*, about 40% of the LCVs were positive for GFP-HDEL two hours after infection (S10A and S10B Fig). In contrast, nearly no GFP-HDEL signals were detected on LCVs in infections with a Dot/Icm deficient strain or the *ΔsidC-sdcA* mutant. While this defect was restored by a plasmid expressing SidC, all the PI(4)P-binding mutants failed to complement the phenotype (S10 Fig). Together, these results demonstrate that P4C-mediated anchoring of SidC to the LCV is critical for the recruitment of ER components to bacterial phagosomes.

## Discussion

**Fig 9:**
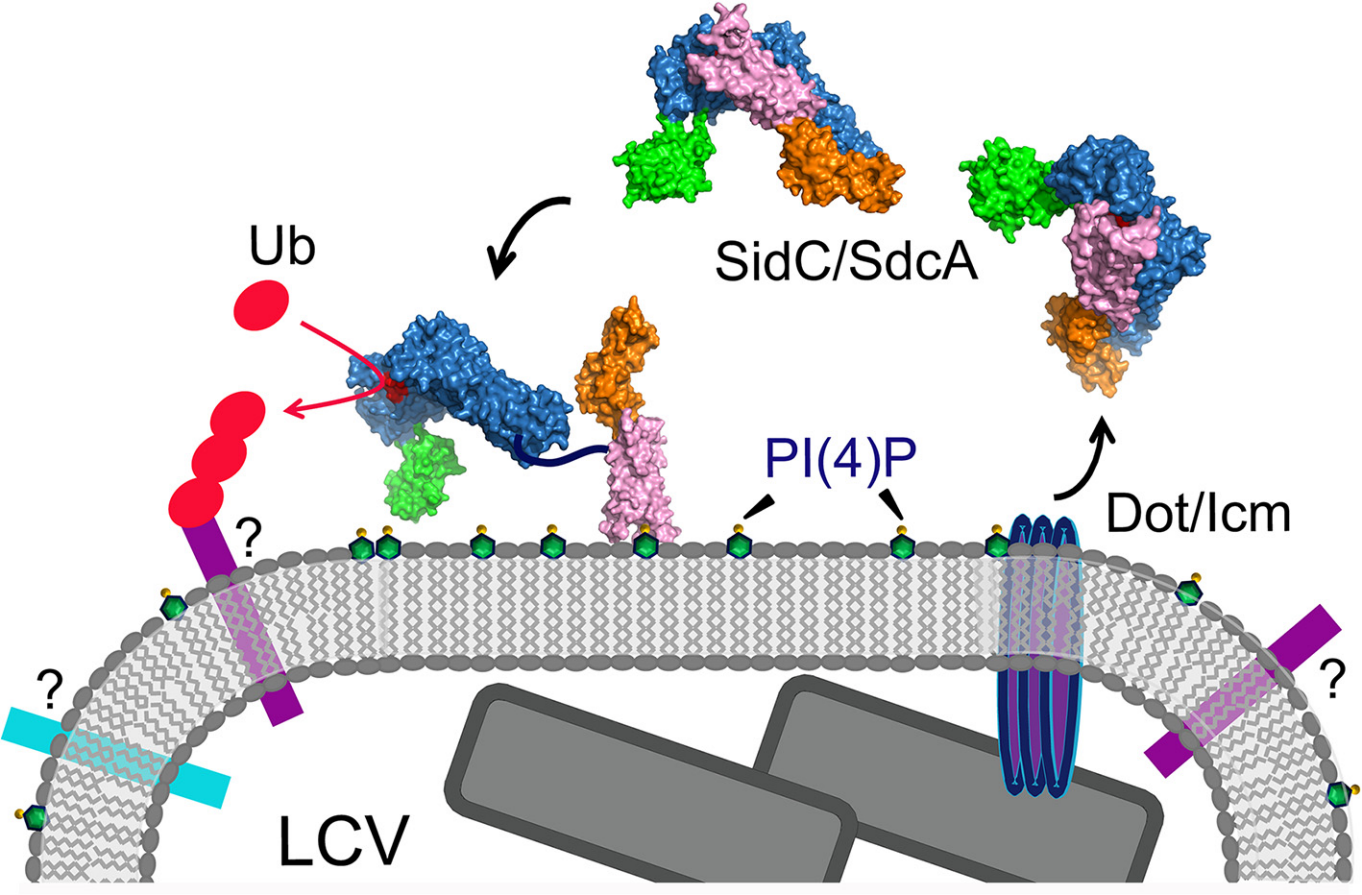
A schematic model of SidC functions at the LCV surface. SidC/SdcA is translocated into the cytosol through the Dot/Icm apparatus. SidC anchors to the LCV membrane through the binding to PI(4)P by its P4C domain. The binding of PI(4)P allows the P4C domain to move away from the SNL domain and exposes the ubiquitin ligase active site. The activated SidC/SdcA ubiquitinates unknown factors, presumably proteins residing on the LCV that determine the identity of the bacterial phagosome.

In this study, we report the structure of the nearly full-length SidC (SidC871), which includes the C-terminal P4C domain. Our structure reveals a novel PI(4)P-binding domain with a four-alpha-helix-bundle fold. The PI(4)P binding site resides at a cationic pocket at one end of the bundle. The P4C domain is packed against the SNL domain and covers the ubiquitin E3 ligase active site (colored in red in Fig 1C). This conformation may represent an inactive closed form of the enzyme. We also show that the L629R mutant switches the enzyme from the closed form to an open conformation and activates the enzyme. We demonstrate that the ubiquitin ligase activity is regulated by PI(4)P. These observations lead us to propose a model for SidC activation on the LCV surface (Fig 9). In this model, SidC/SdcA is translocated into the host in an inactive conformation. The presence of PI(4)P on the LCV allows for high affinity binding via the P4C domain, thus anchoring SidC to the LCV. The binding of PI(4)P triggers a conformational change that extends the P4C domain, which is connected to the SNL domain through a long linker peptide, away from the SNL domain. The separation of the P4C domain from the SNL domain causes the exposure of the ubiquitin ligase active site. This model further implies a mechanism for the tight control of the ubiquitin E3 ligase activity of SidC. It is likely that SidC is active only when it is properly anchored to the surface of the LCV, which allows specific ubiquitination of proteins in the vicinity of SidC. However, the nature of substrates specifically ubiquitinated by SidC/SdcA remains as an interesting question. Since SidC/SdcA is involved in the recruitment of ER-derived vesicles to the LCV, factors that regulate host ER-related membrane trafficking are likely candidates for ubiquitination by SidC/SdcA. The identification of SidC/SdcA substrates will not only aid in understanding the functional role of bacterial virulent effectors, but will also open potential new avenues for the study of host membrane trafficking.

Compared to the previously reported N-terminal structure of SidC [34], our new structure also shows a hinge motion between the INS domain and the SNL domain. This hinge motion is reminiscent of a similar flexible motion between the N- and C-lobes of the HECT family of ubiquitin ligases [37,38]. We hypothesized that the INS domain may directly mediate the binding of E2. Indeed, in our size exclusion chromatography analysis, stable ubiquitin-charged UbcH7 co-fractionated with the wild type N-terminal segment of SidC (1–542), but not with its INS domain-deleted counterpart. These results suggest that the INS domain mediates the interaction of SidC with E2s. This argument also explains our previous observations that SidC and SdcA prefer different E2s for their ligase activity [34]. SidC and SdcA are highly homologous at the primary sequence level, except in the INS domain region. The sequence variability of the INS domain is suggestive of the discriminative binding of E2s by SidC and SdcA. However, how SidC/SdcA bind to their preferred E2s remains to be investigated. The E3 ligase IpaH from *S*. *flexneri* has been shown to bind to E2s at a unique site that is not used by mammalian E3s [52]. Future studies will pursue a structural complex of SidC with ubiquitin-charged UbcH7 to dissect the molecular mechanism for the interaction between SidC/SdcA with E2s.

Our *Legionella* infection experiments show that the binding of PI(4)P via the P4C domain is required for the anchoring of SidC to, and its function on, the LCV. Although the mutants defective for PI(4)P binding still contain an intact ubiquitin E3 ligase domain, they have lost the ability to facilitate the recruitment of ubiquitinated species and ER proteins to the LCV. These results indicate that proper spatial localization of some effectors is crucial for their function during infection. In agreement with this notion, reducing the levels of PI(4)P on the LCV, which likely interferes with proper effector anchoring, has been shown to impair intracellular bacterial replication [29]. Mutations in either the cationic pocket or the MIM that totally abrogate PI(4)P binding in vitro do not completely abolish the association of SidC with the LCV. Moreover, a mutant lacking all PI(4)P-binding determinants (R652Q/L1/L2) shows no further reduction of LCV association compared to single mutations (S9 Fig). This residual binding of SidC to the LCV can be explained by a possible scenario in which SidC interacts with other LCV associated proteins. In fact, it is common to find that the specific membrane targeting of PI binding proteins is governed by a so-called coincidence detection mechanism [23]. Nevertheless, the residual binding of SidC mutants to the LCV does not appear to play an important role in infection, as it does not restore either the ubiquitin signals or the ER proteins recruited to the bacterial phagosome governed by SidC/SdcA.

PI(4)P has emerged as a direct regulator of several cellular processes, including anterograde trafficking from the Golgi complex [53]. The use of fluorescent protein tags fused to protein domains that recognize PI(4)P in vivo has allowed the mapping of this lipid to multiple cellular organelles, such as the Golgi apparatus, the endosomal system, and the plasma membrane [44,54,55]. A sensitive and unbiased PI(4)P probe is essential for the studies of PI(4)P dynamics in living cells. However, current PI(4)P probes have their limitations. For example, the pleckstrin homology (PH) domain of Four-phosphate-adaptor protein 1 (FAPP1) also interacts with ARF1, a Golgi-localized member of the Ras family of small GTPases, thus biasing its localization towards the Golgi complex [44,56,57]. Similarly, this major caveat is also true for the PH domain of Oxysterol-binding protein (OSBP) [58]. Besides interacting with ARF1, the PH domains of FAPP1 and OSBP also recognize PI(4,5)P_2_ in vitro [58,59]. These drawbacks indicate these PH domain-based PI(4)P sensors are less accurate probes. Recently, a new PI(4)P probe derived from the *Legionella* effector protein SidM (DrrA) was reported [46]. The C-terminal P4M domain has a unique structure and has been shown to bind PI(4)P with strong affinity and specificity [30,31,60]. The in vivo application of this novel probe revealed pools of PI(4)P at Rab7-positive late endosomes/lysosomes beyond the Golgi [46]. Here we provide preliminary evidence that the P4C domain of SidC has a high affinity and specificity for PI(4)P binding. Compared with other commonly used PI(4)P probes, the P4C domain appears to have a stronger affinity for PI(4)P-positive liposomes (S6 Fig). Furthermore, the P4C domain displays high selectivity for PI(4)P (Fig 3). The GFP-tagged P4C domain exhibits a high preference for both the Golgi complex and the plasma membrane, where PI(4)P is enriched. Although more quantitative analysis is warranted to demonstrate the superiority of this novel PI(4)P probe, our data suggest that the P4C domain of SidC has a promising potential to be developed into anl accurate and sensitive in vivo PI(4)P probe.

## Materials and Methods

### Cloning and mutagenesis

PCR products for SidC (aa. 1–871) and P4C of SidC (aa. 614–743) amplified from full-length SidC gene were digested with BamHI and XhoI restriction enzymes and inserted into a pET28a-based vector in frame with an N-terminal His-SUMO tag [34]. To generate bacteria expressing GFP-tagged proteins, the same vector was modified by inserting a GFP tag between the His-SUMO tag and the downstream MCS region. Single or multiple amino acid substitutions of SidC were introduced by in vitro site-directed mutagenesis using oligonucleotide primer pairs containing the appropriate base substitutions. SidC542∆INS was generated by ligating two PCR fragments corresponding to residues 1–224 and 324–542. For mammalian expression, wild type and mutant SidC P4C, the PH domain of OSBP, the PH domain of FAPP1 and the P4M domain of SidM were subcloned into the pEGFP-N1 vector or the modified pEGFP-N1 vector with the *egfp* replaced by the mCherry gene. All constructs were confirmed by DNA sequencing.

### Protein expression and purification

*E*. *coli* Rosetta strains harboring the expression plasmids were grown in Luria-Bertani medium supplemented with 50 μg/ml kanamycin to mid-log phase. Protein expression was induced overnight at 18°C with 0.1 mM isopropyl-B-D-thiogalactopyranoside (IPTG). Harvested cells were resuspended in a buffer containing 20 mM Tris-HCl (pH 7.5), 150 mM NaCl, and were lysed by sonication. Soluble fractions were collected by centrifugation at 18,000 rpm for 30 min at 4°C and incubated with cobalt resins (Golden-Bio) for 1 h at 4°C. Protein-bound resins were extensively washed with the lysis buffer. The His-SUMO tag was cleaved by the SUMO-specific protease Ulp1 to release SidC from the resin. Eluted protein samples were further purified by FPLC size exclusion chromatography. The peak corresponding to the expressed protein was pooled and concentrated to 10 mg/ml in a buffer containing 20 mM Tris, pH 7.5, and 50 mM NaCl. SidC542, SidC542 ∆INS, SidC743, SidC743 L629R, SidC743 R652Q, and SidC743 L1/L2 were purified with a similar protocol to the one described above. Ubiquitin, ubiquitin activating enzyme E1, and hUbcH7C85S were expressed and purified as previously described [34].

### SidC-Ub~UbcH7 ternary complex formation

Catalytically inactive ubiquitin conjugating enzyme E2, hUbcH7C85S was charged with ubiquitin at 37°C for 4 h in the presence of 0.5 μM E1, 20 μM E2, and 25 μM ubiquitin in a reaction buffer containing 50 mM Tris-HCl (pH 8.5), 5 mM MgCl2, 0.5 mM DTT, 50 mM creatine phosphate (Sigma P7396), 3 U/ml of pyrophosphatase (Sigma I1643), 3 U/ml of creatine phosphokinase, and 2.8 mM ATP. After charging, SidC542, SidC542 ∆INS, SidC743, or SidC743 L629R was added to the reaction and incubated at 4°C for 3 h. The final mixture was loaded onto a Superdex 200 size exclusion column (GE Life Sciences). Eluted fractions were analyzed by SDS-PAGE.

### In vitro ubiquitin E3 ligase assay

Time course ubiquitination assays were performed at 37°C in the presence of 80 nM E1, 2.8 μM E2, 1 μM SidC truncations or mutants, and 10 μM ubiquitin in a reaction buffer containing 50 mM Tris-HCl (pH 8.0), 5 mM MgCl2, 0.5 mM DTT, 50 mM creatine phosphate (Sigma P7396), 3 U/ml of pyrophosphatase (Sigma I1643), 3 U/ml of creatine phosphokinase, and 2.8 mM ATP. For ubiquitin consumption assays, SidC proteins were pre-incubated with 15 μl 1mM liposome suspensions containing PC/PS or PC/PS/PI(4)P for 30 min at room temperature before the ubiquitination reactions. All reactions were stopped by the addition of 5X SDS-PAGE loading buffer containing 250 mM BME and separated by SDS-PAGE. All the SDS gels were stained with Coomassie blue dye and the intensity of the ubiquitin protein bands was quantified using an Odyssey Infrared imaging system (LI-COR Biosciences). These assays were repeated in three independent experiments. The percentage of ubiquitin usage was averaged and plotted.

### Crystallization, data collection, and processing

Crystals were grown at room temperature by the hanging-drop vapor diffusion method by mixing 1 μl of protein (10 mg/ml) with an equal volume of reservoir solution containing 20% PEG 8,000, pH 7.3, and 0.1 M HEPES. Sheet-shaped crystals were formed within 2–3 days. The crystals were further optimized by microseeding. Crystals were soaked in a cryo-protection solution containing the reservoir solution with 20% glycerol. Soaked crystals were flash frozen in liquid nitrogen before data collection. Diffraction data sets were collected at the Cornell synchrotron light source, MacCHESS beam line A1. The data sets were indexed, integrated, and scaled with HKL-2000 [61]. The crystal belongs to the space group C2 with cell parameters a = 228.16 Å; b = 83.934 Å; c = 129.4 Å; and α = 90^0^, β = 108.82^0^, γ = 90^0^ (S1 Table). The calculated Matthews coefficient Vm = 2.99 and with 58.92% of solvent in the crystal and two protein molecules in an asymmetric unit [62].

### Structure determination and refinement

The crystal structure of SidC871 was determined by molecular replacement using the SidC542 structure (PDB ID: 4TRH) as the search model with the AMoRe program [63] in the CCP4 suite [64]. Iterative cycles of model building and refinement were carried out with the program COOT [65] and the refmac5 program [66] in the CCP4 suite [64]. The final model was quality checked with the Procheck program [67].

### Liposome preparation, flotation assay, and liposome imaging

Phosphatidic acid (PA), 1-palmitoyl-2-oleoyl-sn-glycero-3-phosphocholine (POPC) and 1-palmitoyl-2-oleoyl-sn-glycero-3-phospho-L-serine (POPS) and di-C16-phophatidylinositol polyphosphates were purchased from Avanti. Unilamellar liposomes were generated from a mixture of 64 μg of POPC (80%), 8 μg PS (10%), and 10 μg (10%) of other phospholipids [PI(3)P, PI(4)P, PI(4,5)P_2_, or PA] with addition of 0.08 μg the near-infrared Dil dye (Invitrogen) to aid the visualization and quantitation of lipids. Following air drying, lipid films were hydrated in 50 mM Tris (pH 7.0) and 150 mM NaCl followed by 1h incubation at 37°C for the spontaneous formation of liposomes.

Liposome flotation assays were performed as previously described [41]. Briefly, 10 μL of liposome suspension was mixed with 2 μg proteins and the mixture was brought to 80 μL with HK buffer (20 mM HEPES PH 7.4, 150 mM KOAc) for liposome binding. After 30 min of incubation, 50 μL of 2.5 M sucrose in HK buffer was added to the binding reaction and mixed thoroughly. 30 μL of the sample was taken out as inputs and 100 μL of the sample was transferred to a Beckman 7x20 mm PC ultracentrifuge tube to form the bottom heavy layer. On top of this layer, 100 μL of 0.75 M sucrose-HK buffer was gently overlaid as the middle layer, followed by 20 μL HK buffer as the top layer. Liposomes were separated from unbound protein by ultracentrifugation at 100,000 rpm for 20 min at 20°C in a TLA-100 ultracentrifuge rotor. 30 μL of sample from the top layer was collected and bound proteins were analyzed by SDS-PAGE. Sample loading was normalized based on lipid recovery as measured by the fluorescence of the Dil dye. Western blots were then performed with anti-GFP antibody (1:5,000) to detect the GFP-tagged proteins. Fluorescence intensity of the protein bands was quantified using an Odyssey Infrared imaging system (LI-COR Biosciences). Floatation assays were repeated in three independent experiments. The protein recovery ratios were averaged and plotted.

For liposome imaging, 20 μL of ten-fold diluted liposome suspension was mixed with 20 μL protein solutions containing 0.4 μg proteins for liposome binding. After a 20 min incubation at RT, 5μL of the reaction mixture was added to the chamber created between a cover slip and a glass slide. Fluorescence microscopy images were acquired using a Zeiss Observer inverted microscope (Carl Zeiss) and were analyzed using the Zen software (Carl Zeiss). Fluorescence intensity of the liposomes was quantified using ImageJ. The quantification of the fluorescence intensities were calculated and averaged from three randomly selected liposomes.

### Cell culture, transfection, and fluorescence microscopy

N2A (ATCC) or Cos7 (ATCC) cells were maintained and grown in Dulbecco’s modified Eagle’s medium (DMEM) supplemented with 10% FBS (Cellgro) and 0.1% Pen/Strep (Cellgro) at 37°C in 5% CO2 atmosphere. Mouse bone marrow-derived macrophages were prepared as described [8]. Cells were transfected with 0.5μg of each plasmid by using polyethylenimine (PEI) reagent at a ratio of 1:5. Cells transfected overnight were fixed with 4% formaldehyde. Fluorescence microscopy images were acquired either using a Zeiss Observer inverted microscope (Carl Zeiss) for epi-fluorescence images or a Zeiss LSM 700 confocal microscope (Carl Zeiss) equipped with a 63X oil immersion objective (Carl Zeiss). Images were then analyzed using the Zen software (Carl Zeiss). Fluorescence intensity of cell images was quantified using the ImageJ software. The fluorescence intensities at the cell periphery (for PM) and the perinuclear region (PNR) were integrated. The fluorescence intensity of other areas of the cell was calculated by subtracting the intensities of PM and PNR from the total integrated intensity of the whole cell. The final fluorescence intensities were averaged and plotted from three randomly selected cells.

### *Legionella* strains and infection

*Legionella pneumophila* strains used were Lp02 [68], Lp03 [68] defective for the Dot/Icm secretion system and Lp02∆*sidC/sdcA*, which lacks both *sidC* and *sdcA* [34]. Bacteria were grown on N-(2-acetamido)-2-aminoethanesulfonic acid (ACES)-buffered charcoal-yeast extract agar (CYE) or on CYE supplemented with 100 μg of thymidine/ml (CYET) when necessary [69]. The mutations were introduced into *sidC* on plasmid pZL199 [49]. All mutants were verified by DNA sequencing analysis. The plasmids were transformed into strain Lp02∆*sidC/sdcA*. For infection, bacteria were grown to post-exponential phase as judged by motility and by growth phase (OD600 = 3.4–3.80). An MOI of 1 was used for infections with the U937 human macrophages and for mouse bone marrow-derived macrophages. The *Dictyostelium discoideum* strain AX4 stably expressing GFP-HDEL for assessing the ER membrane conversion phenotype was grown and maintained as previously described [51]. *D*. *discoideum* seeded on glass coverslips in MLB medium [51] was infected with properly grown bacteria at an MOI of 10. One hour after infections, infected cells were washed three times with PBS to remove extracellular bacteria and samples fixed after incubation for an additional hour. *L*. *pneumophila* was labeled with specific antibodies [70] before immunostaining with the FK1 antibody (BIOMOL International/Affiniti, Exeter, United Kingdom) [50] or the SidC specific antibody [36]. Prepared samples were mounted onto slides and inspected under a fluorescence microscope (Olympus IX-81) and images were acquired with a CCD camera.

### Analysis of SidC immuno-fluorescence signals

Images were acquired with an upright Nikon Eclipse 90i laser-scanning confocal microscope equipped with a Nikon 60x oil Plan Apo vc objective. The 488 and 561 nm laser lines (Sapphire Coherent) were used to acquire images of SidC and *Legionella* bacteria, respectively. Only cells that contained bacterial vacuoles with overlapping SidC signals were chosen for imaging. At least 50 fields for each of three coverslips (at least 150 cells) were imaged for each condition. To analyze the signal intensity, integrated intensities of the SidC signal within each LCV region were collected from each field and were averaged per coverslip. The three mean values from each coverslip were then averaged to generate a mean of means +/- SEM. Due to the presence of unequal variances between the strains being compared, raw integrated intensities for each vacuole were log-transformed (log[x+1]), and then re-averaged. Data was analyzed with GraphPad Prism 5 software, using one-way ANOVA followed by Dunnett’s post-hoc test to compare all conditions.

### Accession numbers

Atomic coordinates and structure factors for the reported structures have been deposited into the Protein Data Bank under the accession codes 4ZUZ.

### Ethics statement

The experiments involved in primary murine macrophages were performed in strict accordance with the guidelines of the Public Health Services (PHS) Policy on Humane Care and Use of Laboratory Animals. All animal procedures were performed according to a protocol approved by the Purdue Animal Care and Use Committee (protocol number: 04–081).

## Supporting Information

S1 Fig Representative electron density map. Electron density of the area of the ubiquitin ligase catalytic site is contoured at 1σ after the final cycle of refinement.
(TIF)

S2 Fig Ramachandran plot of residues at the ubiquitin ligase catalytic site. (A) Ramachandran angles of the catalytic site residues (42–47) from the SidC871 structure. (B) Ramachandran angles of the catalytic site residues (42–47) from the SidC542 structure. Note the peptide flipping in residues L42, T45, and C46.
(TIF)

S3 Fig In vitro ubiquitination assays with SidC542 and SidC542ΔINS. Polyubiquitinated species accumulated in reactions with SidC542, however, polyubiquitinated protein bands are absent in reactions with Sidc542ΔINS.
(TIF)

S4 Fig Multiple sequence alignment of the P4C domains of SidC. The sequences corresponding to the P4C domain of SidC (aa. 613–742) from different Legionella species were aligned by Clustal Omega [Sievers F, Wilm A, Dineen D, Gibson TJ, Karplus K, et al. (2011) Fast, scalable generation of high-quality protein multiple sequence alignments using Clustal Omega. Mol Syst Biol 7: 539] and colored by ALSCRIPT [Barton GJ (1993) ALSCRIPT: a tool to format multiple sequence alignments. Protein Eng 6: 37–40]. Secondary elements are drawn below the alignment. The L1 and L2 loop are marked with squares. Two arginine residues forming the cationic binding pocket are marked by “&”. Hydrophobic residues (W641, W642, F643, W704, and F705) that form the MIM motif are highlighted with “*”. The interface residue L629 is indicated by “#”. Entrez database accession numbers are as follows: SidC_Phili, gi: 52842719; SidC_Lens, gi: 54295348; SidC_Paris, gi: 54298515; SidC_Corby, gi: 148360028; SdcA__Lens, gi: 54295347; SdcA__Phili, gi: 52842718; SdcA__Alcoy, gi: 296108150; SdcA__Corby, gi: 148360029; SdcA__Paris, gi: 54298514; SidC__Longbeach, gi: 289166408.
(TIF)

S5 Fig Intracellular localization of GFP-P4C and its PI(4)P-binding defective mutants in Cos7 cells. Epi-fluorescent images of Cos7 cells transfected with GFP-tagged P4C or PI(4)P-binding defective mutants. The nucleus was stained with DAPI. P4C showed both plasma membrane and perinuclear localization. The R638Q, L1, and L2 mutants had a more diffuse localization, while the R652Q and the L1/L2 mutants were completely cytosolic.
(TIF)

S6 Fig Comparison of PI(4)P-containing liposome binding by PI(4)P probes. (A) Western blot of samples from liposome floatation assays. Recombinant proteins of GFP-P4C, GFP-FAPP1-PH, GFP-P4M, and GFP-OSBP-PH were incubated with PI(4)P-containing liposomes. Proteins that floated with liposomes were analyzed by Western blot using poly-clonal antibodies against GFP. (B) Quantification of liposome floatation assays from three independent assays. Error bars represent standard deviation. (C) Fluorescent images of liposome binding by GFP-tagged probes. The same amount of GFP-fusion proteins were incubated with PI(4)P-containing liposomes. After incubation at room temperature for 20 min, mixtures were applied to glass cover-slips for imaging. The GFP-P4C demonstrated the strongest binding affinity with PI(4)P-containing liposomes. Scale bar = 10 μm. (D) Quantification of liposome binding of GFP-P4C. GFP fluorescent signals were normalized to red Dil dye signals on the same liposome and averaged on three randomly picked liposomes. Error bars represent standard deviation. ** *p* < 0.01; *** *p* < 0.001.
(TIF)

S7 Fig SidC expression and translocation after *Legionella* infection. (A) Expression of SidC and its mutants in *L*. *pneumophila*. Bacteria were grown in ACES-buffered medium to OD_600_ = 3.5 and collected cells were lysed with SDS-PAGE sample buffer. SidC was detected with a specific antibody and the metabolic enzyme isocitrate dehydrolase (ICDH) was probed as a loading control. *Legionella* strains are the same as used in Fig 8. (B) Translocation of SidC mutants by the Dot/Icm transporter. U937 cells were infected with *L*. *pneumophila* strains at an MOI of 2 for 2hrs. Infected cells were lysed with 0.2% saponin and the soluble fractions were probed for SidC after SDS-PAGE. The host protein tubulin was detected as a loading control. *Legionella* strains are the same as used in (A).
(TIF)

S8 Fig The anchoring of SidC to the LCV is dependent on PI(4)P binding by the P4C domain. (A) Immuno-fluorescent staining of SidC on the LCV. Bone marrow-derived macrophages were infected with indicated *L*. *pneumophila* strains at an MOI of 1 for 2hrs. Samples were first stained for extracellular and intracellular bacteria before being stained for SidC with specific antibody. Scale bars, 2 μm. (B) Percentage of cells containing SidC positive LCVs counted from three independent experiments (at least 150 vacuoles were scored in each experiment).
(TIF)

S9 Fig The association of the PI(4)P-binding defective SidC mutants with the LCV are significantly reduced. (A) SidC immuno-fluorescent signal intensity analysis on SidC-positive vacuoles. U937 cells were infected with L. pneumophila strains at an MOI of 2 for 2hr. Samples were stained for bacterium (red) and SidC (green). Images were acquired and SidC signals were processed and analyzed as described in Materials and Methods. (B) SidC immuno-fluorescent signal intensities from wild type and PI(4)P-binding defective mutants were plotted. In addition to the significant reduction of SidC-positive LCVs (S8 Fig), the intensity of SidC immuno-fluorescent signals on SidC-positive vacuoles are also decreased by about 10 fold when infected by Legionella strains expressing PI(4)P-binding defective SidC mutants. Experiments were done in triplicate and at least 150 vacuoles were scored for each treatment.
(TIF)

S10 Fig PI(4)P-binding by the P4C domain is involved in the ER marker recruitment to the bacterial phagosome. (A) Images show the recruitment of the ER marker GFP-HDEL (green) to the LCVs in *D*. *discoideum* cells infected with the indicated *Legionella* strains (red). Scale bars, 2 μm. Legionella strains are the same as used in S7 and S8 Figs. (B) Percentage of cells containing GFP-HDEL positive LCVs counted from three independent assays under the conditions infected with the indicated *Legionella* strains.
(TIF)

S1 Table Describes the parameters for x-ray data collecting, processing, and structure refinement. (DOCX)
