# Supplementary figures and images for: Structure of the Legionella Virulence Factor, SidC Reveals a Unique PI(4)P-Specific Binding Domain Essential for Its Targeting to the Bacterial Phagosome
Source: PLoS Pathog. 2015 Jun 12;11(6):e1004965. doi: 10.1371/journal.ppat.1004965 (PMC4467491; doi:10.1371/journal.ppat.1004965)

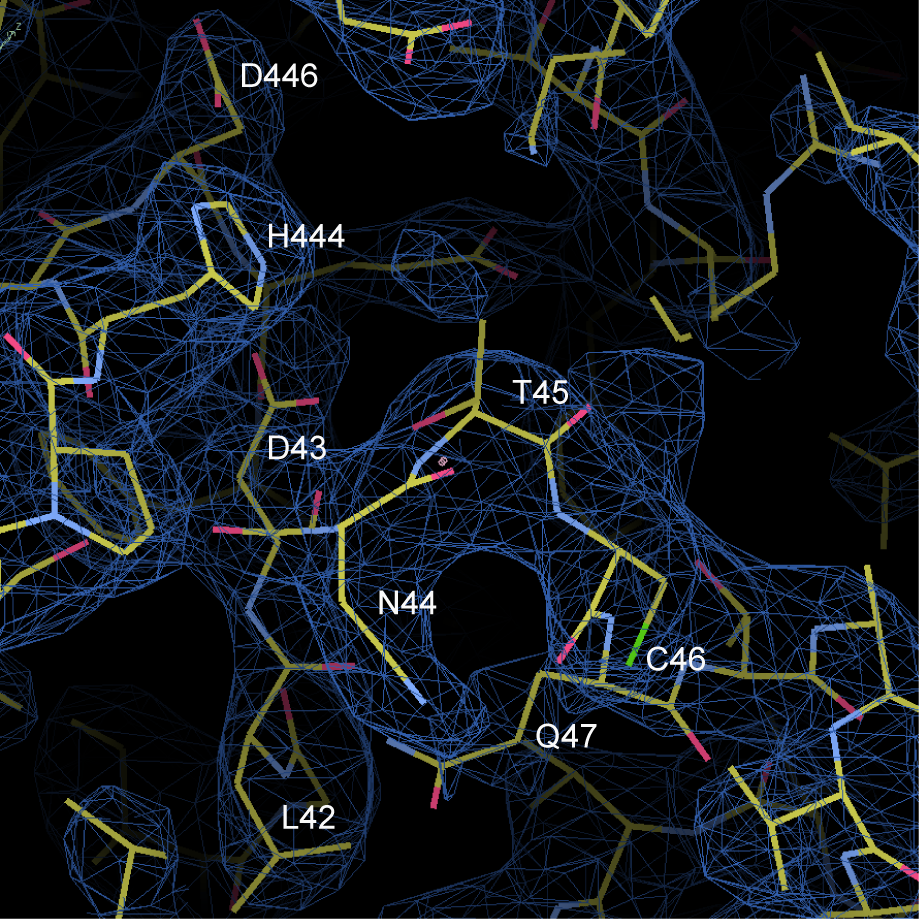

Supplement: S1 Fig — Electron density of the area of the ubiquitin ligase catalytic site is contoured at 1σ after the final cycle of refinement. (TIF) [file ppat.1004965.s001.tif]

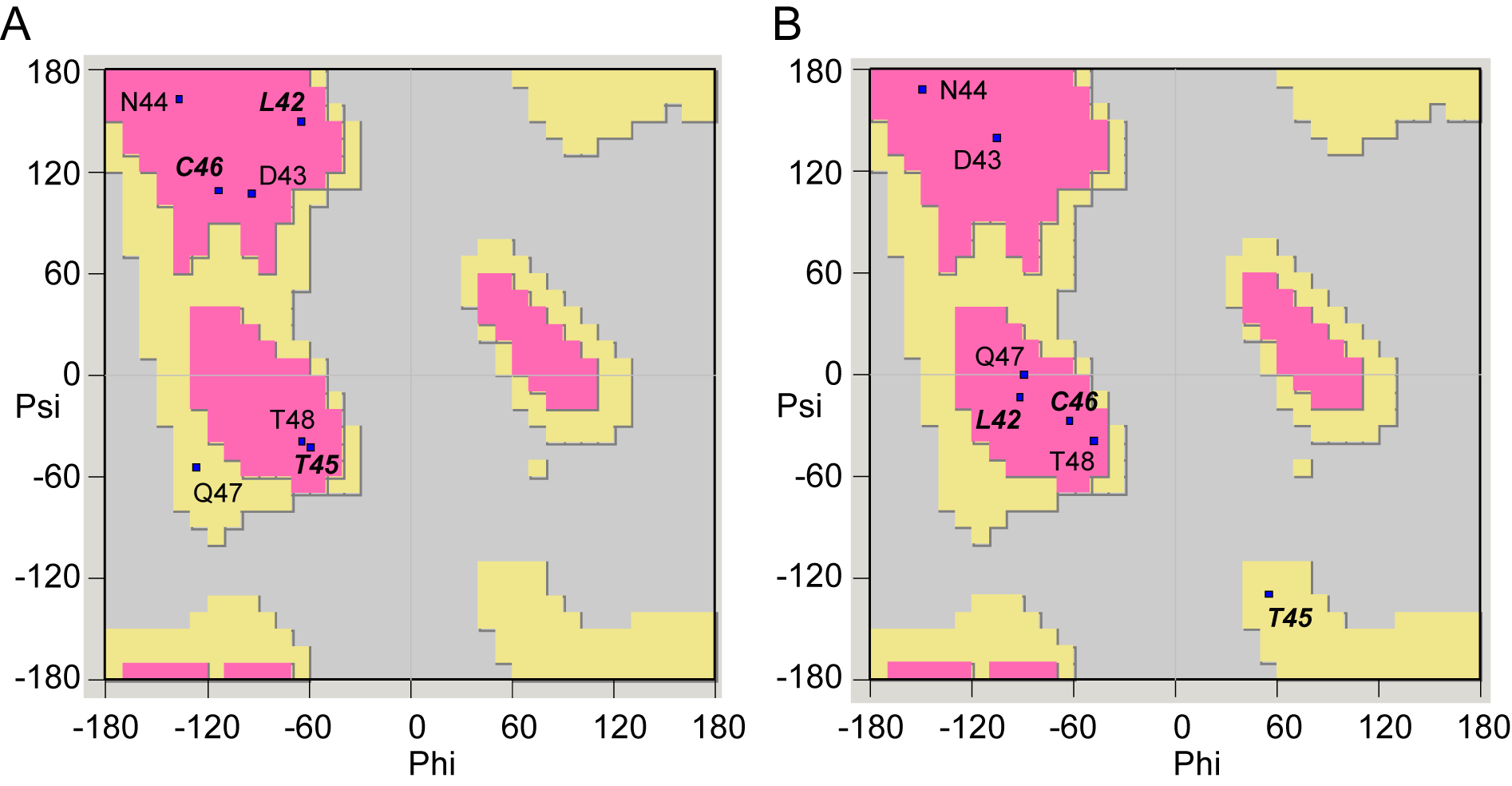

Supplement: S2 Fig — (A) Ramachandran angles of the catalytic site residues (42–47) from the SidC871 structure. (B) Ramachandran angles of the catalytic site residues (42–47) from the SidC542 structure. Note the peptide flipping in residues L42, T45, and C46. (TIF) [file ppat.1004965.s002.tif]

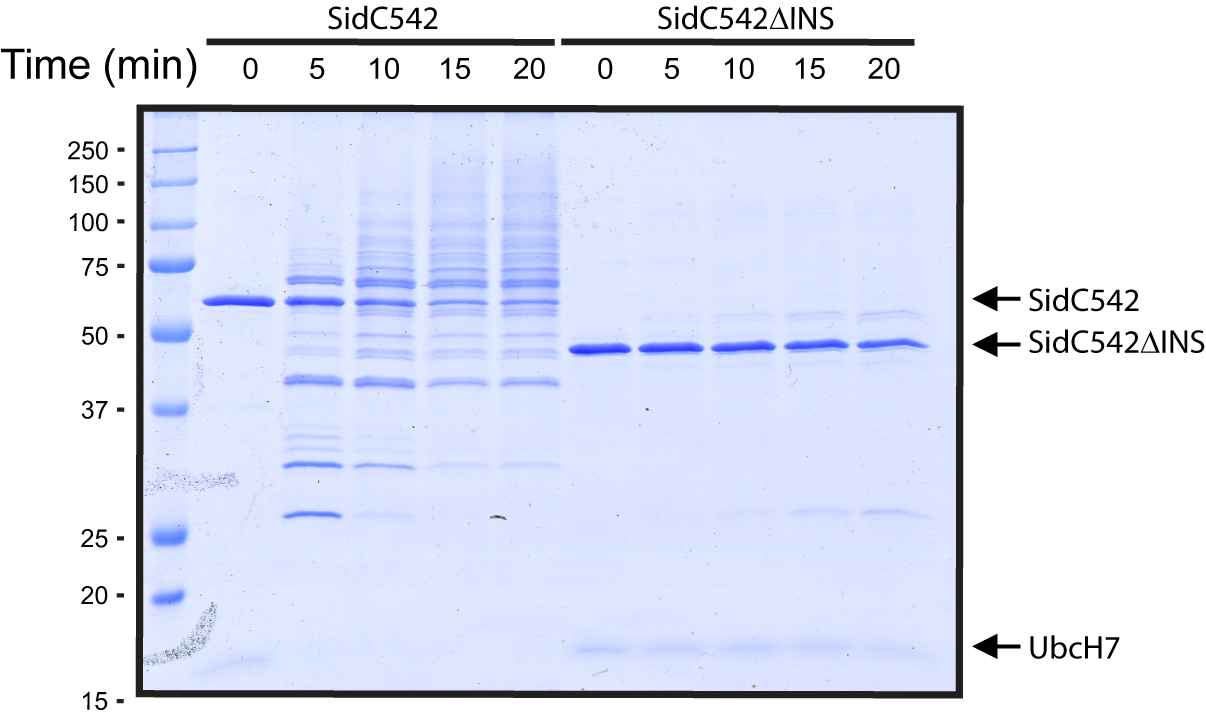

Supplement: S3 Fig — Polyubiquitinated species accumulated in reactions with SidC542, however, polyubiquitinated protein bands are absent in reactions with Sidc542ΔINS. (TIF) [file ppat.1004965.s003.tif]

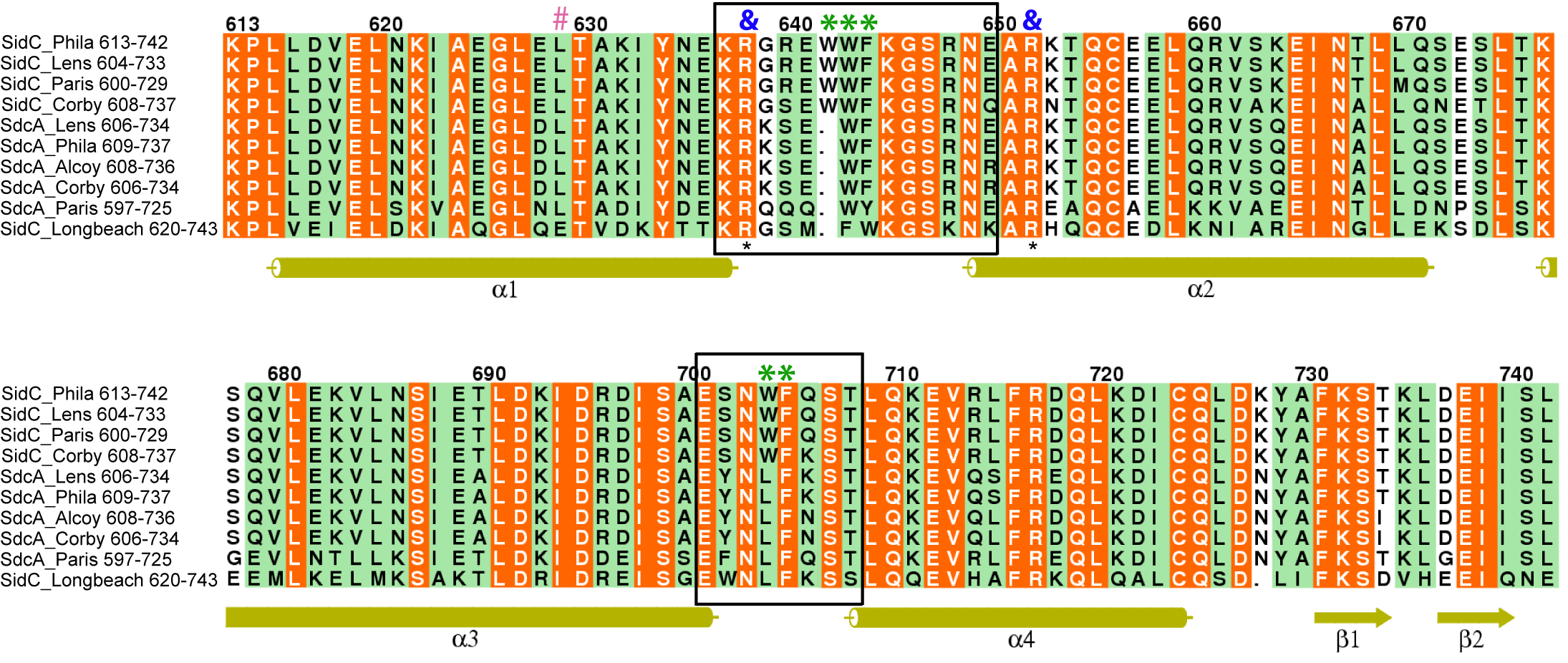

Supplement: S4 Fig — The sequences corresponding to the P4C domain of SidC (aa. 613–742) from different Legionella species were aligned by Clustal Omega [Sievers F, Wilm A, Dineen D, Gibson TJ, Karplus K, et al. (2011) Fast, scalable generation of high-quality protein multiple sequence alignments using Clustal Omega. Mol Syst Biol 7: 539] and colored by ALSCRIPT [Barton GJ (1993) ALSCRIPT: a tool to format multiple sequence alignments. Protein Eng 6: 37–40]. Secondary elements are drawn below the alignment. The L1 and L2 loop are marked with squares. Two arginine residues forming the cationic binding pocket are marked by “&”. Hydrophobic residues (W641, W642, F643, W704, and F705) that form the MIM motif are highlighted with “*”. The interface residue L629 is indicated by “#”. Entrez database accession numbers are as follows: SidC_Phili, gi: 52842719; SidC_Lens, gi: 54295348; SidC_Paris, gi: 54298515; SidC_Corby, gi: 148360028; SdcA__Lens, gi: 54295347; SdcA__Phili, gi: 52842718; SdcA__Alcoy, gi: 296108150; SdcA__Corby, gi: 148360029; SdcA__Paris, gi: 54298514; SidC__Longbeach, gi: 289166408. (TIF) [file ppat.1004965.s004.tif]

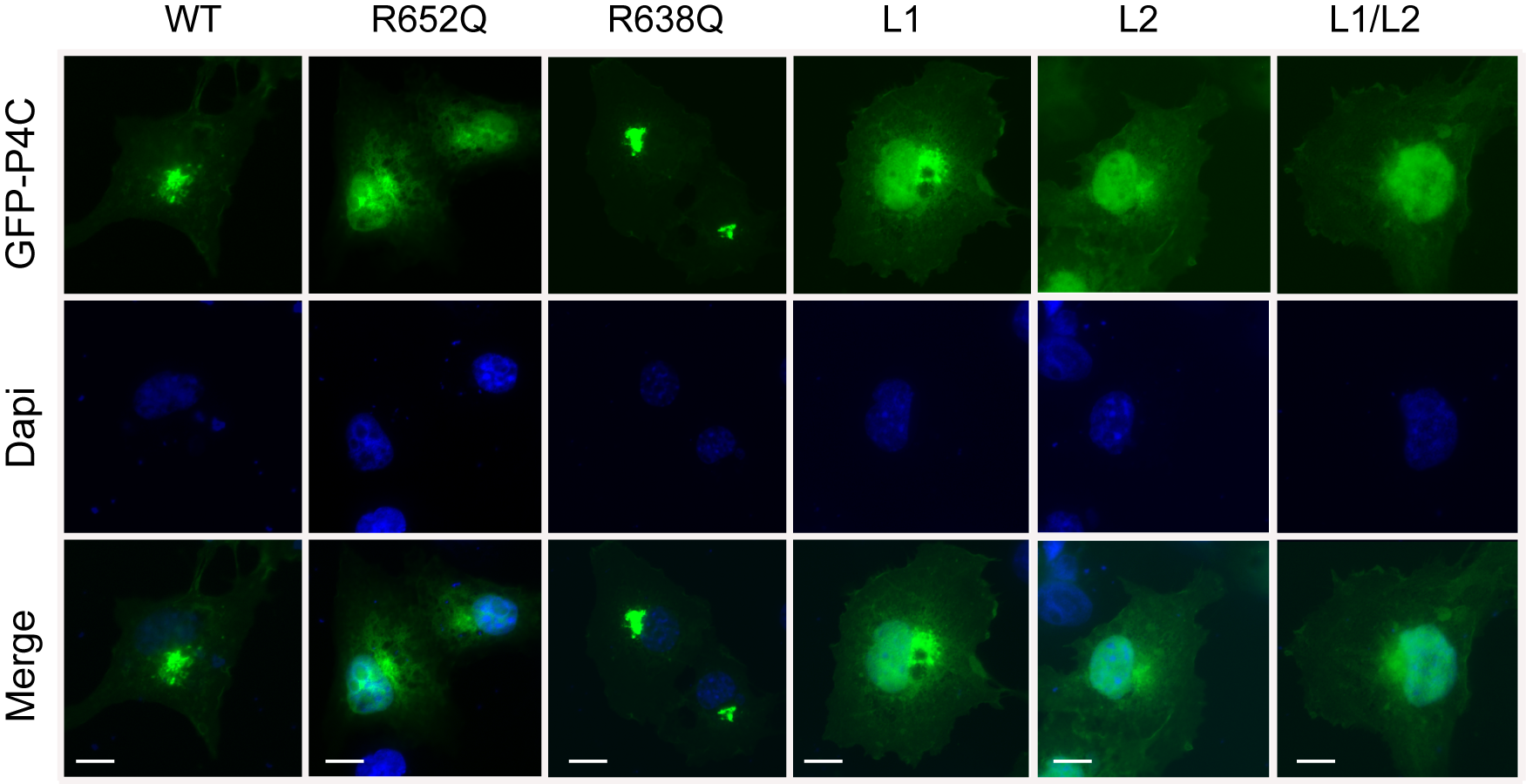

Supplement: S5 Fig — Epi-fluorescent images of Cos7 cells transfected with GFP-tagged P4C or PI(4)P-binding defective mutants. The nucleus was stained with DAPI. P4C showed both plasma membrane and perinuclear localization. The R638Q, L1, and L2 mutants had a more diffuse localization, while the R652Q and the L1/L2 mutants were completely cytosolic. (TIF) [file ppat.1004965.s005.tif]

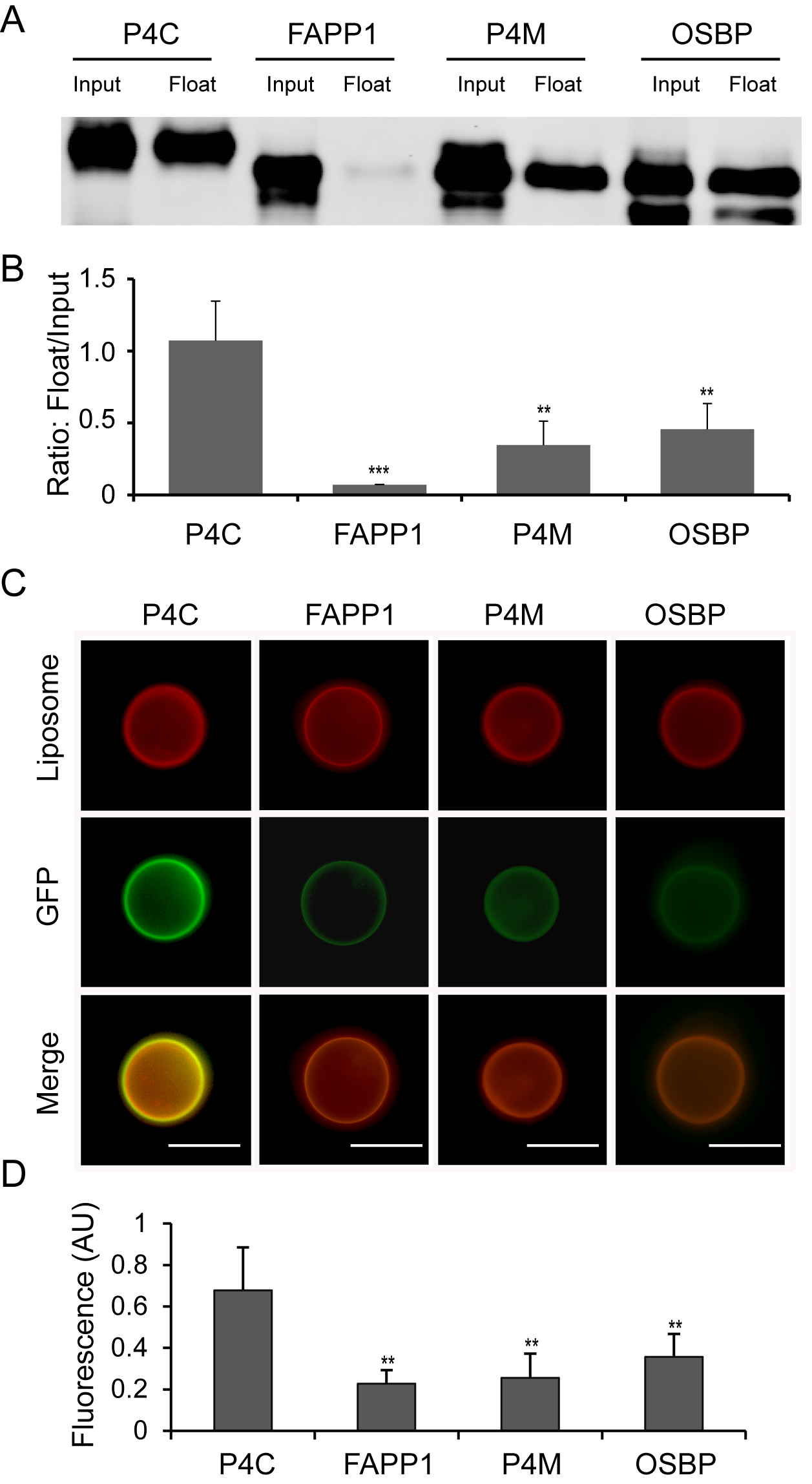

Supplement: S6 Fig — (A) Western blot of samples from liposome floatation assays. Recombinant proteins of GFP-P4C, GFP-FAPP1-PH, GFP-P4M, and GFP-OSBP-PH were incubated with PI(4)P-containing liposomes. Proteins that floated with liposomes were analyzed by Western blot using poly-clonal antibodies against GFP. (B) Quantification of liposome floatation assays from three independent assays. Error bars represent standard deviation. (C) Fluorescent images of liposome binding by GFP-tagged probes. The same amount of GFP-fusion proteins were incubated with PI(4)P-containing liposomes. After incubation at room temperature for 20 min, mixtures were applied to glass cover-slips for imaging. The GFP-P4C demonstrated the strongest binding affinity with PI(4)P-containing liposomes. Scale bar = 10 μm. (D) Quantification of liposome binding of GFP-P4C. GFP fluorescent signals were normalized to red Dil dye signals on the same liposome and averaged on three randomly picked liposomes. Error bars represent standard deviation. ** p < 0.01; *** p < 0.001. (TIF) [file ppat.1004965.s006.tif]

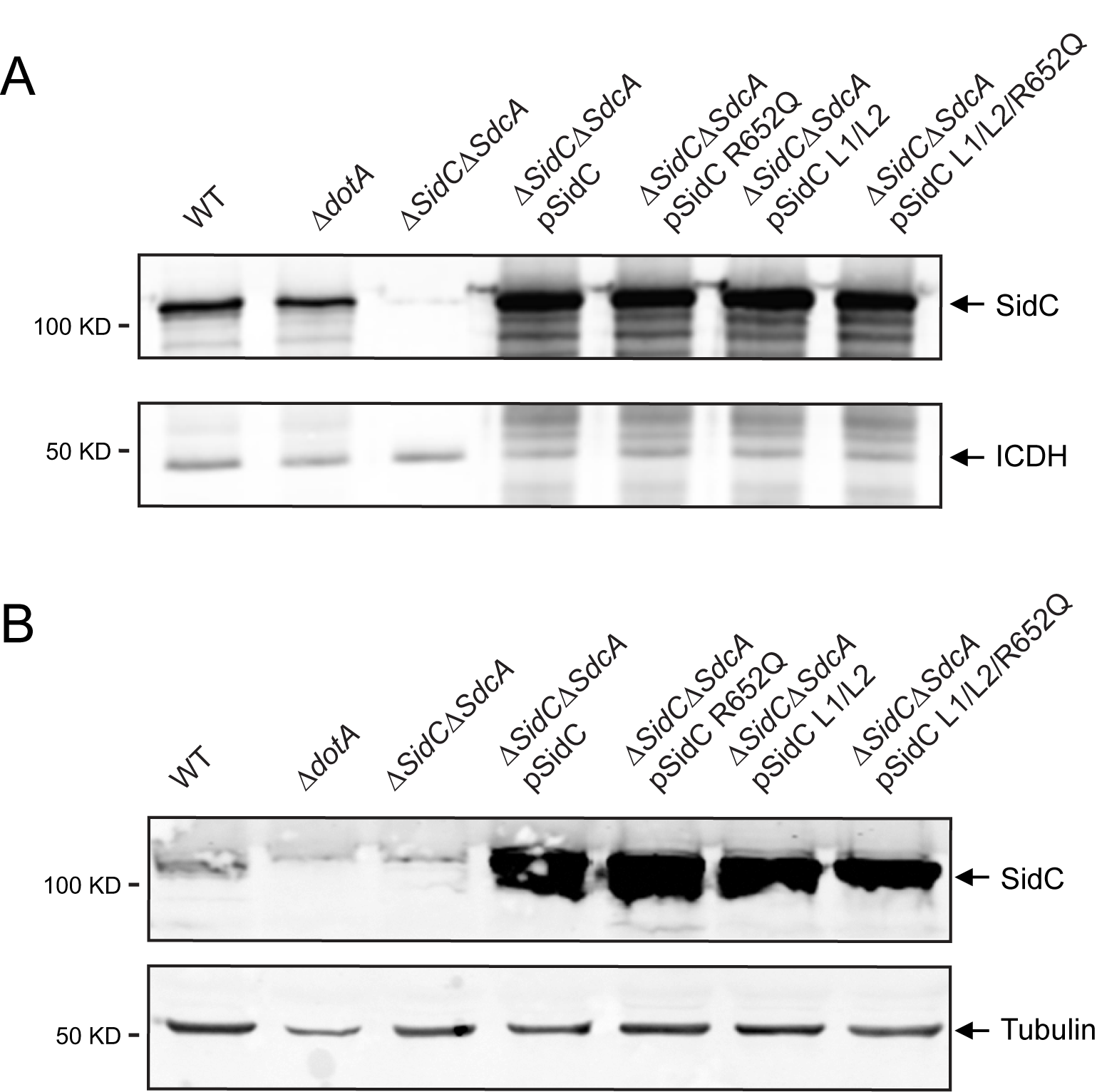

Supplement: S7 Fig — (A) Expression of SidC and its mutants in L. pneumophila. Bacteria were grown in ACES-buffered medium to OD600 = 3.5 and collected cells were lysed with SDS-PAGE sample buffer. SidC was detected with a specific antibody and the metabolic enzyme isocitrate dehydrolase (ICDH) was probed as a loading control. Legionella strains are the same as used in Fig 8. (B) Translocation of SidC mutants by the Dot/Icm transporter. U937 cells were infected with L. pneumophila strains at an MOI of 2 for 2hrs. Infected cells were lysed with 0.2% saponin and the soluble fractions were probed for SidC after SDS-PAGE. The host protein tubulin was detected as a loading control. Legionella strains are the same as used in (A). (TIF) [file ppat.1004965.s007.tif]

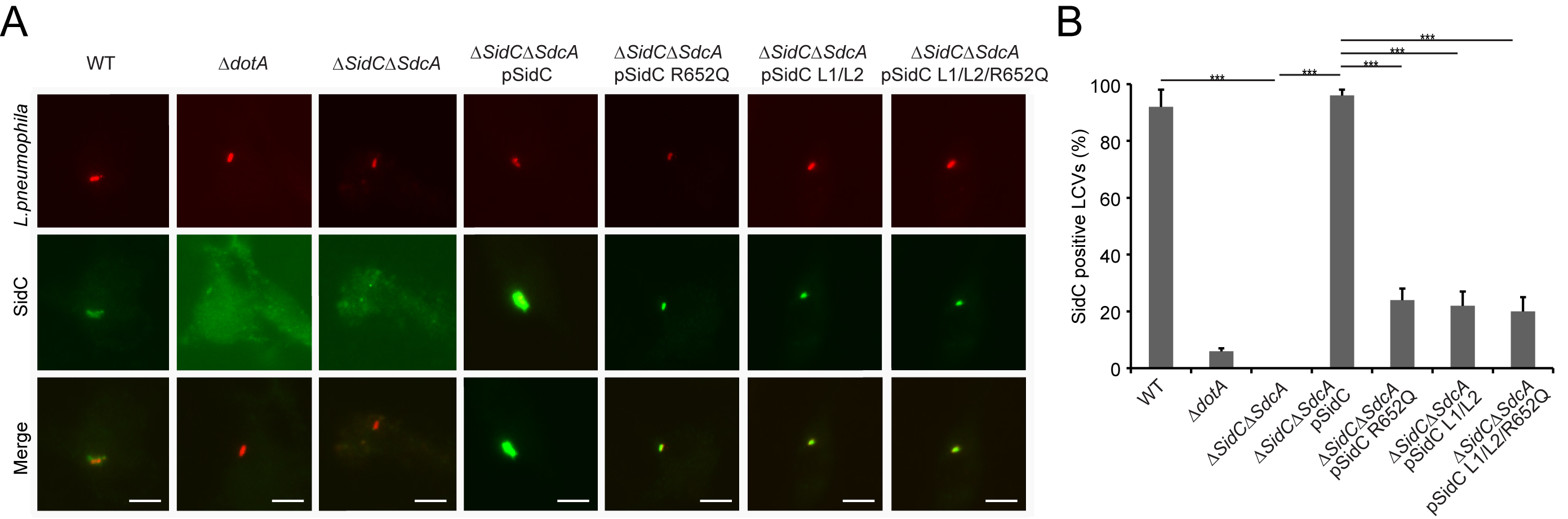

Supplement: S8 Fig — (A) Immuno-fluorescent staining of SidC on the LCV. Bone marrow-derived macrophages were infected with indicated L. pneumophila strains at an MOI of 1 for 2hrs. Samples were first stained for extracellular and intracellular bacteria before being stained for SidC with specific antibody. Scale bars, 2 μm. (B) Percentage of cells containing SidC positive LCVs counted from three independent experiments (at least 150 vacuoles were scored in each experiment). (TIF) [file ppat.1004965.s008.tif]

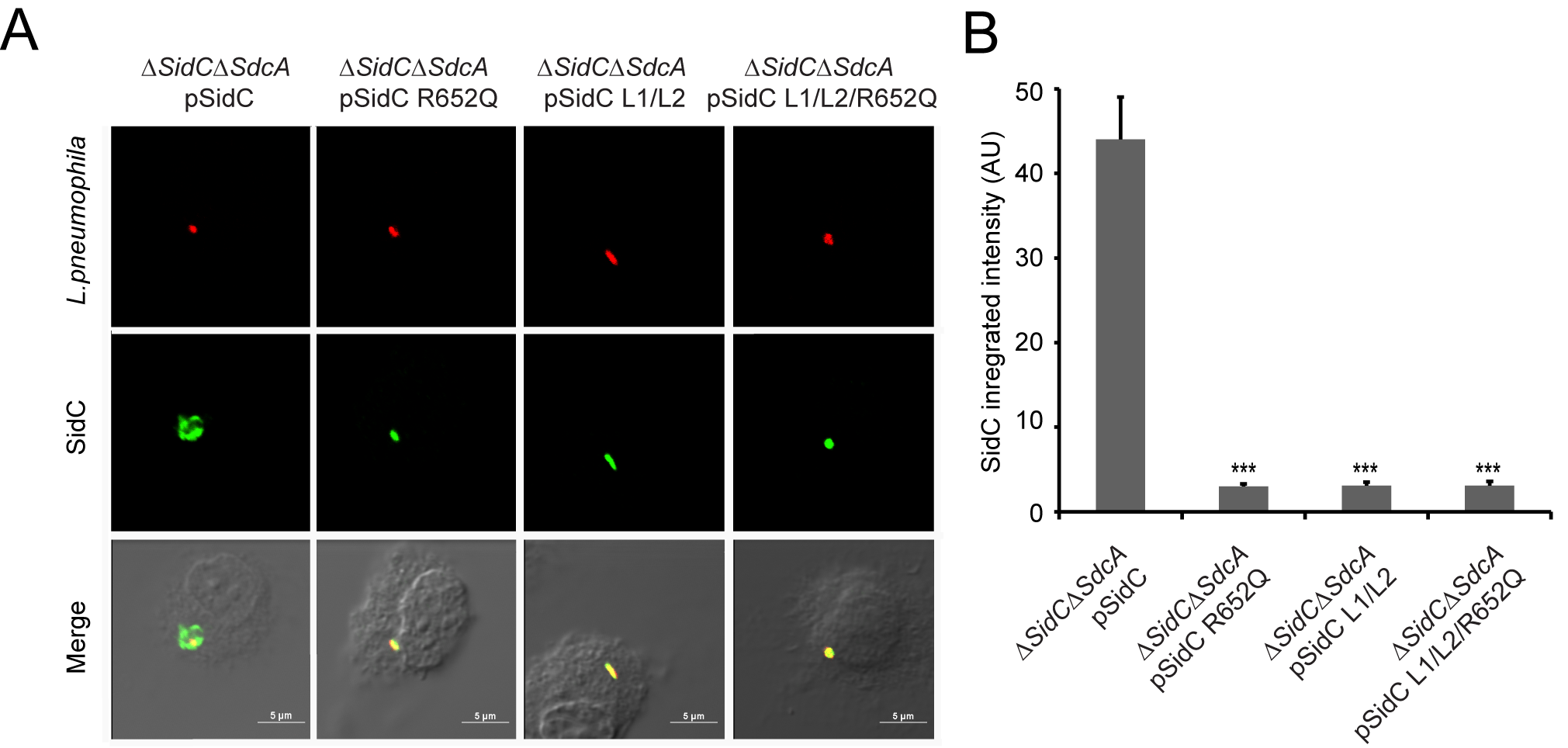

Supplement: S9 Fig — (A) SidC immuno-fluorescent signal intensity analysis on SidC-positive vacuoles. U937 cells were infected with L. pneumophila strains at an MOI of 2 for 2hr. Samples were stained for bacterium (red) and SidC (green). Images were acquired and SidC signals were processed and analyzed as described in Materials and Methods. (B) SidC immuno-fluorescent signal intensities from wild type and PI(4)P-binding defective mutants were plotted. In addition to the significant reduction of SidC-positive LCVs (S8 Fig), the intensity of SidC immuno-fluorescent signals on SidC-positive vacuoles are also decreased by about 10 fold when infected by Legionella strains expressing PI(4)P-binding defective SidC mutants. Experiments were done in triplicate and at least 150 vacuoles were scored for each treatment. (TIF) [file ppat.1004965.s009.tif]

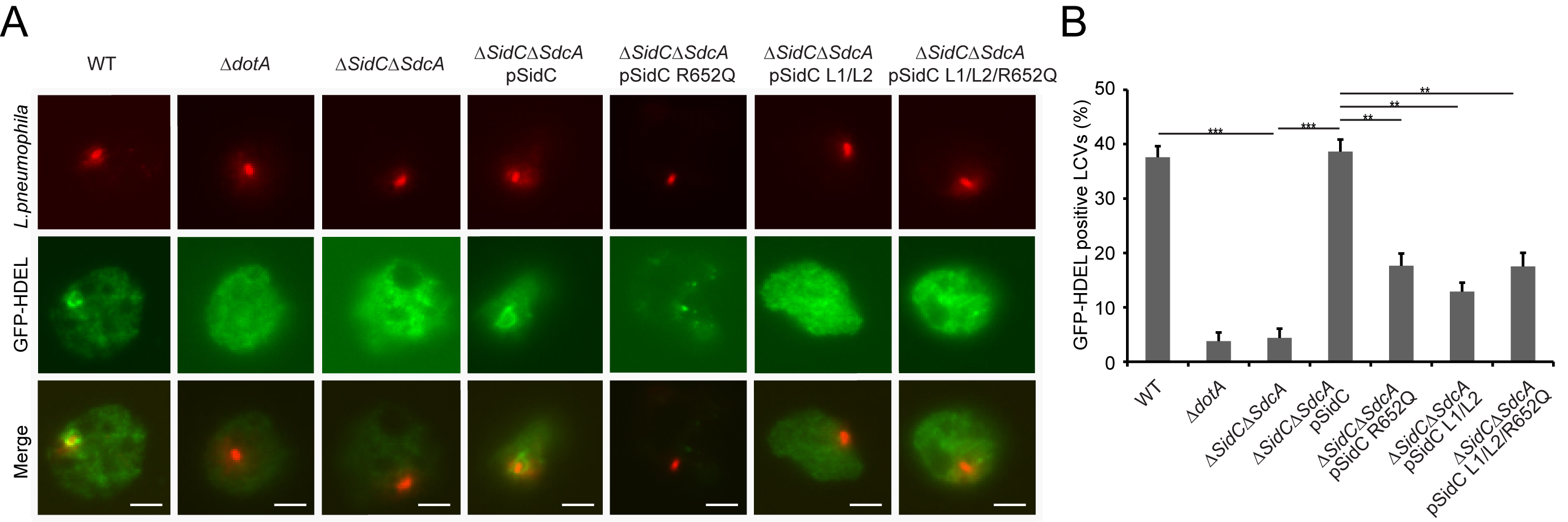

Supplement: S10 Fig — (A) Images show the recruitment of the ER marker GFP-HDEL (green) to the LCVs in D. discoideum cells infected with the indicated Legionella strains (red). Scale bars, 2 μm. Legionella strains are the same as used in S7 and S8 Figs. (B) Percentage of cells containing GFP-HDEL positive LCVs counted from three independent assays under the conditions infected with the indicated Legionella strains. (TIF) [file ppat.1004965.s010.tif]
