# Supplementary material for: Structure of the Legionella Virulence Factor, SidC Reveals a Unique PI(4)P-Specific Binding Domain Essential for Its Targeting to the Bacterial Phagosome
Source: PLoS Pathog. 2015 Jun 12;11(6):e1004965. doi: 10.1371/journal.ppat.1004965 (PMC4467491; doi:10.1371/journal.ppat.1004965)
Supplement: S1 Table — (DOCX) [file ppat.1004965.s011.docx]

**Supplemental Table S1.**  Data collection, phasing and structural refinement statistics

| A. Data collection statistics | | |
| --- | --- | --- |
| Space group | C2 | |
| Cell dimensions | a = 228.16 Å, b = 83.934 Å, c = 129.4 Å, α = 90^0^, β = 108.82^0^, γ = 90^0^ | |
|  | Native | |
| Synchrotron beam lines | MCCHESS A1 | |
| Wavelength (Å) | 0.9759 |  |
| Maximum resolution (Å) | 2.86 | |
| Observed reflections | 266,725 | |
| Unique reflections | 53,345 | |
| Completeness (%)^a^ | 100.0(99.9) | |
| <I>/<σ>^a^ | 46.5(2.7) | |
| R_sym_^a,b^ (%) | 9.0(79.0) | |
| B. Refinement statistics | Native | |
| Resolution (Å)^a^ | 50-2.86(3.01-2.86) | |
| R_crys_ / R_free_ (%)^a,c^ | 22.0/28.3(38.5/44.5) | |
| Rms bond length (Å) | 0.01 | |
| Rms bond angles (°) | 1.24 | |
| Ramachandran plot |  | |
| Most favored/Additional (%) | 95.0/5.0 | |
| Generous/Disallowed (%) | 0/0 | |
| ^a^Values in parenthesis are for the highest resolution shell. | | |
| ^b^R_sym_ = Σ_h_Σ_i_\|I_I_(h) − <I(h)\|/Σ_h_Σ_i_I_I_(h). | | |
| ^c^R_crys_ = Σ(\|F_obs_\|−k\|F_cal_\|)/Σ\|F_obs_\|. R_free_ was calculated for 5% of reflections randomly excluded from the refinement. | | |
